# The effects of chemical interactions and culture history on the colonization of structured habitats by competing bacterial populations

**DOI:** 10.1186/1471-2180-14-116

**Published:** 2014-05-07

**Authors:** Simon van Vliet, Felix JH Hol, Tim Weenink, Peter Galajda, Juan E Keymer

**Affiliations:** 1 Department of Bionanoscience, Kavli Institute of Nanoscience, Delft University of Technology, Lorentzweg 1, Delft, CJ 2628, The Netherlands; 2 Department of Environmental Systems Science, ETH Zurich, Universitätsstrasse 16, 8092 Zürich, Switzerland; 3 Department of Environmental Microbiology, Eawag, Überlandstrasse 133, Dübendorf 8600, Switzerland; 4 Institute of Biophysics, Biological Research Centre of the Hungarian Academy of Sciences, Temesvari krt. 62, Szeged H-6726, Hungary; 5 Instituto de Ecología y Biodiversidad (IEB), Las Palmeras 3425 Ñuñoa, Santiago, Casilla 653, Chile; 6 Current address: Department of Bioengineering, Imperial College London, London, UK

**Keywords:** Habitat colonization, Spatially structured habitats, Microbes, Collective behavior, Bacterial competition, Microfluidics

## Abstract

**Background:**

Bacterial habitats, such as soil and the gut, are structured at the micrometer scale. Important aspects of microbial life in such spatial ecosystems are migration and colonization. Here we explore the colonization of a structured ecosystem by two neutrally labeled strains of *Escherichia coli*. Using time-lapse microscopy we studied the colonization of one-dimensional arrays of habitat patches linked by connectors, which were invaded by the two *E. coli* strains from opposite sides.

**Results:**

The two strains colonize a habitat from opposite sides by a series of traveling waves followed by an expansion front. When population waves collide, they branch into a continuing traveling wave, a reflected wave and a stationary population. When the two strains invade the landscape from opposite sides, they remain segregated in space and often one population will displace the other from most of the habitat. However, when the strains are co-cultured before entering the habitats, they colonize the habitat together and do not separate spatially. Using physically separated, but diffusionally coupled, habitats we show that colonization waves and expansion fronts interact trough diffusible molecules, and not by direct competition for space. Furthermore, we found that colonization outcome is influenced by a culture’s history, as the culture with the longest doubling time in bulk conditions tends to take over the largest fraction of the habitat. Finally, we observed that population distributions in parallel habitats located on the same device and inoculated with cells from the same overnight culture are significantly more similar to each other than to patterns in identical habitats located on different devices inoculated with cells from different overnight cultures, even tough all cultures were started from the same −80°C frozen stock.

**Conclusions:**

We found that the colonization of spatially structure habitats by two interacting populations can lead to the formation of complex, but reproducible, spatiotemporal patterns. Furthermore, we showed that chemical interactions between two populations cause them to remain spatially segregated while they compete for habitat space. Finally, we observed that growth properties in bulk conditions correlate with the outcome of habitat colonization. Together, our data show the crucial roles of chemical interactions between populations and a culture’s history in determining the outcome of habitat colonization.

## Background

Bacteria use sophisticated mechanisms to sense, predict and respond to environmental changes in time and across space. Chemotaxis directs the movement of individual cells towards their likes (attractants) and away from their dislikes (repellents) while quorum sensing and cell-signaling help bacteria coordinate their behavior at the population level [1-5]. Bacteria growing together in a common location actively change their surroundings by depleting nutrients, producing metabolites, and secreting signaling-molecules [2,6,7]. This collective conditioning of the environment, combined with the individual response of cells to their changing environment, can lead to the formation of complex patterns in spatiotemporal cell distributions [7,8].

In spatially structured habitats, migration and colonization are important features of population dynamics. In his classic work, Adler showed that *Escherichia coli* can spread on agar plates as traveling population waves [2,6]. The formation and migration of these waves is driven by chemotaxis along gradients in nutrient concentration, bacteria form these gradients as they consume nutrients [2,6]. Moreover, on plates initially lacking any chemoattractants, both *E. coli* and *Salmonella typhimurium* can form symmetrical patterns consisting of spots and rings, caused by chemotaxis towards self-secreted attractants [7-10]. Many species, including *E. coli*, can also form complex patterns consisting of branching colony structures [11-15]. Despite the fact that such colony development is influenced by a myriad of environmental factors, regularities in these patterns have been described [16-19].

Previous studies that illuminated important aspects of microbial life in spatial environments used habitats (i.e. agar plates) that lack fine spatial structure. However, natural environments of bacteria such as soil [20-23] and the gut [24-26] have structure at multiple spatial scales, including the micrometer to millimeter range. In these heterogeneous (patchy) environments, metapopulations (i.e. local populations coupled by migration) are likely to develop [27]. Recently, microfluidic devices have become a powerful tool to study bacteria in such spatially structured environments. Microfluidic devices have been used to study the behavior of single cells within collectively moving populations [28-31] and the effects of spatially structured habitats [32-35] and heterogeneously distributed nutrients [36,37] on population dynamics.

Most work so far has studied a single population colonizing a new habitat. However, in natural systems different populations can invade habitats from multiple locations. Previous work using agar plates has shown how interactions between populations originating from separate inoculations of the same strain can lead to the development of complex macroscopic patterns [2,19,38-41] however, a microscopic investigation of these interactions is currently lacking.

Here, we investigate the invasion of spatially structured habitats by two separate populations in microscopic detail. Time-lapse fluorescence microscopy of two differentially labeled strains of *E. coli* allows us to resolve dynamics within the interacting populations down to the single cell level. In order to approximate the natural patchy environment of bacteria, we make use of microfabrication to create spatially structured habitats, consisting of coupled arrays of habitat patches. We focus on three related questions (i) how are these patchy habitats colonized? (ii) how do the two strains invading from opposite ends of the landscape interact during the colonization of the habitat? and (iii) how reproducible are the colonization patterns?

We found that cells colonize a habitat from opposite sides by a series of traveling waves followed by an expansion front. The populations invading from opposite ends do not mix in the habitat, rather, colonization waves collide and expansion fronts compete for the landscape. We demonstrate that these interactions are mediated by diffusible chemicals. We found that the qualitative features of the colonization patterns are similar for all experiments, even though population distributions vary widely between experiments. However, when parallel habitats located on the same device are inoculated from the same initial cultures, we observe strikingly similar population distributions.

## Results

**Figure 1:**
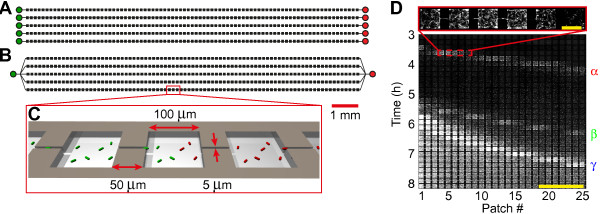
**Colonization of spatially structured synthetic ecosystems. (A)** Device of type-1 with 5 parallel habitats (habitats 1 to 5 from top to bottom), each consisting of 85 patches, with separate inlets. Red cells are inoculated on the right (indicated by red inlet holes) and green cells on the left (green inlet holes). **(B)** Device of type-2 with a single, shared, inlet. Except for the inlet, devices in **A** and **B** are identical. **(C)** Enlarged schematic view of the devices shown in **A** and **B** showing an array of patches of 100 × 100 × 5 μm^3^ linked by connectors of 50 × 5 × 5 μm^3^. Note that the bacteria are not to scale. **(D)** Kymograph of fluorescence intensity of the left most 25 patches for strain JEK1036 (green) showing a typical pattern of landscape invasion consisting of three subsequent colonization waves (α at *t* ≈ 3.5 h, β at *t* ≈ 5 h and γ at *t* ≈ 6 h) followed by the expansion front (at *t* ≈ 6 h); scale bar = 1 mm. The inset at the top shows an enlarged view of the α wave just after entering the habitat from the inlet; scale bar = 100 μm.

Using microfabrication we created devices consisting of five parallel habitats, each consisting of an array of 85 patches connected by narrow connectors (Figure 1A-C). Habitats are connected to either individual inlets (type 1 devices, Figure 1A), or to a single shared inlet (type 2 devices, Figure 1B) used for inoculation. Unless noted otherwise, two differentially labeled, but otherwise isogenic, strains of *E. coli* were inoculated at opposite sides of the habitats. We refer to cells and populations of these strains as ‘green’ (strain JEK1036) and ‘red’ (strain JEK1037). The neutrality of the two markers was demonstrated in previous work [42] and verified here by measuring growth in bulk conditions (see Methods and Additional file 1).

### Colliding waves decompose into distinct components

After inoculation, the populations initially grow in the inlet holes and start to colonize the habitats after 2 to 4 hours. During the first phase of colonization typically three waves enter the habitat, as can be seen in Figure 1D. The first two waves (α and β) are of relatively low cell density (≈500 cells per wave), while the third wave (γ) is a high-density wave at the leading edge of an expansion front (Figure 1D). In most (32 out of 48) habitats, three waves with densities and velocities similar to Figure 1D are seen for at least one of the two strains, while in all 48 habitats (on 11 devices of types-1 and 2, see Additional files 2 and 3) at least a single wave is observed. These colonization waves require chemotaxis, as a smooth-swimming, non-chemotactic, *cheY* knockout strain did not form any waves (Additional file 4A). Bacteria in a wave remain tightly packed while traveling throughout the patchy habitat, although there is some limited dispersion of the wave profile (Additional file 5). The observed wave profiles (Additional file 5A-C) and velocities (<*v*>=0.86 μm/s, Additional file 5D) compare well to those described in previous work, where wave velocities of 1.8 to 3.8 μm/s were reported for linear channels [29,30,43], while waves in large unstructured chambers traveled at 0.56 μm/s [33]. This indicates that a patchy spatial structure does not interfere with the formation and propagation of bacterial population waves. Interestingly, the waves span multiple (roughly 5) patches, indicating that traveling populations are formed at scales larger than that of the habitat patches.

**Figure 2:**
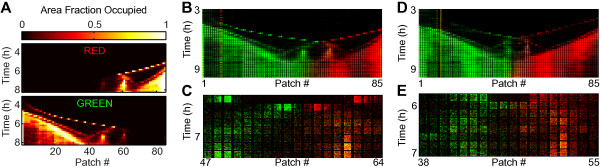
**The collisions of colonization waves. (A)** Occupancy measure (area fraction) calculated per patch for strains JEK1037 (red) and JEK1036 (green) showing the collision between two α waves (at *t* = 6 h, patch 54). Note how both waves branch: a part of the wave is reflected, a part forms a stationary population, and a part continuous (for a short distance) in the same direction. **(B)** Kymograph of fluorescence intensity for the collision shown in **A**. **(C)** Enlarged view of **B**, centered at the point of collision. Note how the red and green populations remain largely segregated in space, even though individual cells do mix with the other population. **(D)** Kymograph of fluorescence intensity of a collision in a different habitat in the same device (with separate inlets; type-2) as the habitat shown in **A-C**. Note the similarity between **B** and **D**. **(E)** Enlarged view of **D**, centered at the point of collision.

**Figure 3:**
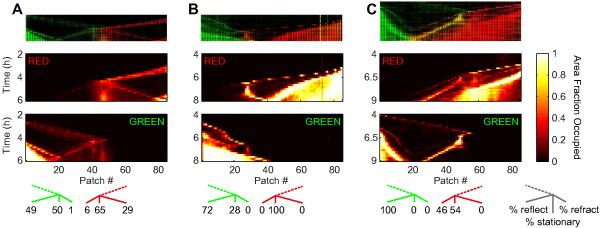
**Decomposition of colliding colonization waves.** The top row shows kymographs of fluorescence intensity, the second row shows occupancy levels for strain JEK1037 (red), the third row the occupancy levels for strain JEK1036 (green), and the bottom row the post-collision distributions of bacteria over the reflected, stationary and refracted components (from left to right for green and from right to left for red), as determined from the occupancy distribution 1 hour after the collision. Examples where: **(A)** Both waves have large reflected parts. **(B)** Red wave forms a stationary population. **(C)** Most of the red wave is refracted. Also note how a combined wave (yellow, in top row) is formed when the red β wave collides with a stationary green population (*t* = 6.5 h, patch 50).

When two waves coming from opposite inlets collide, they give rise to complex but reproducible spatiotemporal patterns (Figure 2). Figure 2A shows data depicting a green wave coming from the left and a red wave coming from the right. After their collision, most green cells remain grouped with other green cells, either in the reflected wave traveling back towards the left inlet, or in a large stationary population (Figure 2A, *t* = 7 h). The red cells show a similar post-collision distribution, consisting of a reflected wave and a stationary population spatially separated from their green counterpart (Figure 2A). As most cells stay with their original population, it is still possible to distinguish between ‘red’ and ‘green’ populations after the collision. Notice, however, that a small number of cells do mix into the other population as can be seen in patch 60 in Figure 2C, for example. It is interesting to note that the reflected wave reverses direction within 10 minutes, without first forming a detectable stationary subpopulation, contrary to previous observations where reflected waves reverse direction on much longer time scales (~1 h), after first forming a stationary population [38]. We observe similar collision patterns between colonization waves even when both sides of the habitat are inoculated with cells from the same strain, indicating that these collisions are not an artifact of the fluorescent markers (Additional file 4B-D).We observe that patterns of wave collisions are similar in habitats on the same device (i.e. habitats inoculated with cells from the same set of initial cultures; compare Figure 2B with D and C with E), however, there is a large variation in the collision patterns between habitats on different devices inoculated with cells from a different set of initial cultures (Figure 3). For each wave the post-collision outcome can be decomposed in three components: (i) part of the wave is reflected back, continuing to travel as a wave after quickly (within 10 min) having reversed its direction; (ii) part of the wave disintegrates and a local (sessile) population is formed; (iii) part of the wave is ‘refracted’, continuing to travel as a wave in the same direction as before the collision, although typically with a lower velocity. The distribution of bacteria from the incoming wave over these three components can vary strongly between devices, as can be seen in Figure 3. For example: in Figure 3A the green and red α-waves both have strong reflected parts (49% and 29% of the cells in the red and green α-waves, respectively), in Figure 3B the red α-wave completely disintegrates and in Figure 3C a large part (46%) of the red α-wave is refracted. The patterns can become more complex if subsequent incoming waves interact with the subpopulations formed in the initial collision. For example in Figure 3C, a red β-wave merges with a green stationary populations and a combined, two-strain wave (yellow), is formed and starts traveling to the left of the habitat.

### Incoming expansion fronts remain spatially segregated

**Figure 4:**
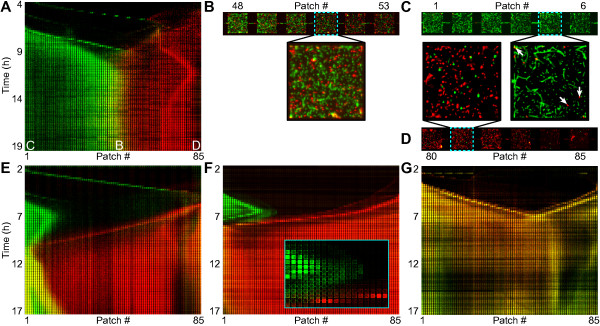
**Interactions between expansion fronts. (A)** Kymograph of fluorescence intensity for a habitat where a stable boundary is observed. **(B)** Enlarged view of panel **A**, for the 6 patches centered at the interface between the green and red populations at *t* = 19 h. **(C)** Enlarged view of the 6 patches at the left end of the habitat shown in **A** at *t* = 19 h. A few red cells are indicated by the white arrows in the inset. **(D)** Enlarged view of the 6 patches at the right end of the habitat shown in **A** at *t* = 19 h. **(E)** Kymograph of fluorescence intensity where the green population is expelled from the habitat by the red population, before the two fronts come into physical contact. **(F)** Kymograph of fluorescence intensity where the green population is expelled from the habitat by the red population, the inset shows that there has not been any physical contact between red cells and the green front before the latter changes direction. Note how the leading edge of the green front and the high density region, roughly 8 patches behind the leading edge, change direction almost simultaneously **(G)** Kymograph of fluorescence intensity for a habitat inoculated at both sides with a single mixed culture of strains JEK1036 (green) and JEK1037 (red), note that the change in color from red to green with increasing time is mostly due to changes in the fluorescence intensity per cell of RFP compared to GFP and not due to de-mixing of the strains as can be seen by comparing their occupancy patterns (Additional file 7A).

Following the colonization waves, two expansion fronts enter the habitat from opposite ends (Figures 1D and 4). Upon encountering each other, these fronts form a boundary that exhibits a gradual transition from a majority of green cells to a majority of red cells over a distance of 5 to 10 patches (Figure 4A,B and Additional files 2 and 3). Except for this relatively narrow transition zone, the two strains remain spatially segregated over the course of the experiment. However, individual cells do move across the entire habitat (Figure 4C,D) suggesting that there is no physical barrier for cells to cross the boundary.

In 4 out of 11 devices (of type-1 and 2) the boundary between the two expansion fronts remains in the same location (e.g. Figure 4A). However, in the other cases (7 out 11) the location of the boundary shifts over time and one of the populations eventually occupies at least two-thirds of the habitat (e.g. Figure 4E,F and Additional files 2 and 3). On average both strains take over the habitat an equal number of times indicating that they are neutral when averaged over many experiments (Additional file 6 and Methods). To confirm this, we inoculated a device on both sides with cells from a 1:1 mixed culture of the two strains. The habitats are colonized by waves and expansion fronts consisting of a mixed (‘yellow’) community of the two strains (Figure 4G). Over the course of the experiment both strains remained mixed both on the local (patch) and global (habitat) scale with a high degree of overlap in the spatial distribution of the two strains (Additional file 7), showing that the two strains are neutral when growing in patchy habitats. Furthermore, this shows that when the same two strains are cultured and inoculated separately they remain spatially segregated, while if they are cultured and inoculated together, they remain mixed.

We further investigated whether the success of a strain in the structured habitats, measured as the area fraction of the habitat that they occupy (i.e. their occupancy), can be predicted from their growth in batch culture. To do so, we investigated the relation between growth properties of the initial cultures and the occupancy obtained in the habitat. We found that there is a significant positive correlation between the relative doubling times of the two initial cultures in bulk and the relative occupancies they obtain in the habitat (*r*^2^ = 0.36, *p* = 0.002, Pearson correlation, analyzed for *t* = 18 h, Additional file 6C). This indicates that the slowest growing culture (*i.e.* the culture with the longest doubling time) in bulk conditions tends to colonize the largest part of the habitat. It should be noted that both strains have similar doubling times and can obtain a majority fraction of the habitat (see Methods). This suggests that although the two strains are neutral when averaged over many experiments, in each individual experiment small differences between the initial cultures translate into different outcomes of the colonization process. We observe a similar trend when looking at the occupancy averaged over the entire colonization process (Additional file 6B) while there are no, or only weak, effects of other properties of the initial cultures (such as their optical density, see Additional file 6A).Interestingly, one strain can drive the other out of the habitat before their expansion fronts physically meet: Figure 4E shows a green population front that reverses direction shortly after (≈1 h) a red population front entered the habitat from the right, even though the two fronts are separated by approximately 1 cm at that time. Figure 4F shows a green population that stops and reverses direction before a single cell of the red population has reached the green front (Figure 4F inset).

### Interactions between populations are chemically mediated

**Figure 5:**
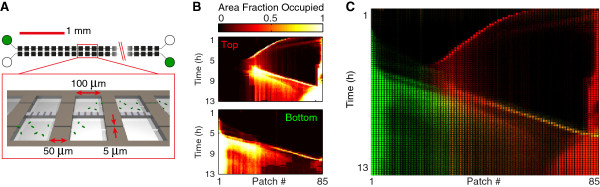
**Interactions between chemically coupled, but physically separated populations. (A)** Schematic of a microfabricated device of type-3, consisting of two parallel habitats (each of 85 patches) chemically coupled by 200 nm deep nanoslits of 15 × 15 μm, which allow for the diffusion of molecules but are too shallow for bacteria to pass through. **(B)** Area fraction occupied per patch (occupancy) for the top and bottom habitats, the top habitat is inoculated from the right and the bottom habitat from the left with the same initial culture of strain JEK1036 (green). **(C)** Kymograph where the fluorescence intensities of the top and bottom habitats are superimposed: cells in the top habitat are shown in red and cells in the bottom habitat in green. Note that both habitats are inoculated from the same (JEK1036) culture and that the bacteria in the upper and lower habitats are spatially confined to their own habitat.

As a consequence of the observations described above, we hypothesized that chemical interactions (e.g. gradients in nutrients, metabolites, signaling-molecules etc.) but not physical interactions (e.g. spatial exclusion) are the main mechanisms underlying the collisions of colonization waves as well as the interactions between expansion fronts. We believe so for three reasons: (i) wave collisions occur even at low cell densities (≈500 cells per wave), (ii) populations remain spatially segregated even though cells could pass freely across the boundary, and (iii) two fronts interact over large distances or when they are separated by vacant patches. To test this hypothesis, we designed a third type of device (type-3) consisting of two parallel, diffusionally coupled arrays of patches (Figure 5A). These two habitats are coupled by 200 nm deep nanoslits, which allow for the diffusion of nutrients, metabolites and signaling molecules while being too shallow for bacteria to pass through [44], thereby confining each metapopulation to a single habitat.

The two coupled habitats were inoculated from top-left and bottom-right ends with cells from the same initial culture (of JEK1036, Figure 5A). Figure 5B and C show that ‘collisions’ of waves and expansion fronts also occur between these physically separated, but chemically coupled clonal populations. For example, the wave in the top habitat coming from the right (Figure 5B,C, red) stopped and formed a stationary population when it reached the (low density) wave coming from the left in the bottom habitat (Figure 5B,C, green). Furthermore, the high-density regions in the top and bottom habitats remain largely segregated in space (Figure 5C and Additional file 8), indicating that even though these populations inhabit two different habitats they still avoid being close to one another. Thus demonstrating the importance of chemical interactions in structuring the spatiotemporal distribution of bacterial populations.

### The degree of similarity between population distributions is influenced by the initial culture

**Figure 6:**
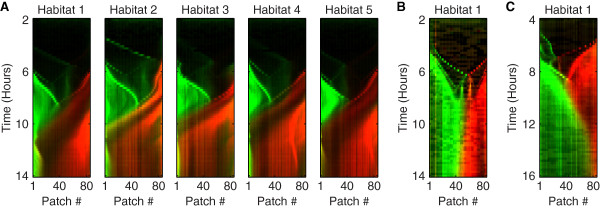
**Similarity of spatiotemporal patterns for habitats inoculated with same cultures.** Kymographs show the fluorescence intensity of strains JEK1036 (green; inoculated from the left at *t* = 0 h) and JEK1037 (red; inoculated from the right at *t* = 0 h). **(A)** Five parallel habitats in the same device (type 1) with separate inlets, each kymograph shows the spatiotemporal pattern of a single habitat. **(B)** Habitat on a different device inoculated with a different set of initial cultures (with separate inlets; type-1) than in panel **A**. **(C)** Habitat in a device (type-2) with a shared inlet. Note the similarity between the patterns of the five habitats in panel **A** (all inoculated with the same initial cultures), compared to the patterns of the habitats in panels **B** and **C** (inoculated with different cultures than the habitats in **A**).

We observed that the population distribution in habitats on the same device, which were inoculated with cells coming from the same set of initial cultures, are highly similar to each other (e.g. compare the five habitats in Figure 6A). Even in the early phases of colonization, when there are only about a thousand cells present in the entire habitat, patterns are similar to each other (e.g. compare Figure 2B and D and see Additional files 2 and 3 for all data). Conversely, we observed a large variation between the population distributions in habitats located on different devices that were inoculated with cells coming from different sets of initial cultures (e.g. compare Figure 6A with 6B or C).

We performed a quantitative analysis to investigate whether there is a significant difference in the degree of similarity between habitats located on the same device, which were inoculated from the same cultures, compared to habitats located on different devices, which were inoculated from different cultures. The similarity of patterns was quantified by calculating the difference between the patterns using eq. 1 (Methods), which ranges from *d* = 0 for identical patterns to *d* = 1 for maximally different patterns. We found that the average difference between the population distributions in habitats located on the same device and inoculated from the same set of initial cultures (*d*_*same*_) is significantly smaller than the average difference between patterns of habitats inoculated with different sets of initial cultures (*d*_*different*_, see Additional file 9). This is the case both for devices with independent inlets (24 habitats in 6 type-1 devices, randomization test, *p* < 0.001; <*d*_*same*_*>*=0.28 and *<d*_*different*_*>*=0.38, mean values, see Additional file 9A) as well as for devices with a shared inlet (24 habitats in 5 type-2 devices, randomization test, *p* < 0.001; <*d*_*same*_*>*=0.22 and *<d*_*different*_*>*=0.39, see Additional file 9B).

The similarity of population distributions in habitats in the same device could potentially be caused by a coupling between habitats (*e.g.*, diffusion through the PDMS layer which seals the devices), an identical response of the bacteria to device-wide gradients (*e.g.*, of oxygen or temperature) or by other extrinsic variation. We tested for these possibilities using two sets of experiments. First, we used a type-4 device that consists of two habitats separated by 1.2 mm, which are inoculated in reverse order (red from the left in habitat 1 and from the right in habitat 2, Additional file 10B). The patterns in these two habitats were similar to each other (*d* = 0.28, Additional file 10A), suggesting that spatial proximity is not a necessity for obtaining similar population distributions in replicate habitats.

Secondly, we used devices of type-5 consisting of four parallel habitats, which were inoculated from two sets of initial cultures such that neighboring habitats were colonized by different cultures (see Methods and Additional files 11 and 12). We found that neighboring habitats inoculated from different initial cultures do not become more similar due to their proximity to each other, with a median difference between patterns in habitats located on the same device, but inoculated from different cultures, of *d*_*different*_ = 0.32 (median, 25%-75% quartiles = 0.27-0.42), which is similar to the observed value of the difference between patterns in habitats located on separate type-1 and 2 devices, which were inoculated from different cultures, of *d*_*different*_ = 0.38 (median, 25%-75% quartiles = 0.37-0.40; *p* = 0.32, Wilcoxon rank sum test, N = 8 for type-5 devices, N = 10 for type-1 and 2 devices combined, Additional file 9C). This demonstrates that population distributions in neighboring habitats that were inoculated from the same initial cultures are not similar just because of their location next to each other on the same device.

For the type-5 devices the difference between habitats inoculated from different initial cultures is calculated by comparing habitats on the same device, while for the type-1 and 2 devices this difference is calculated by comparing habitats located on different devices. To make sure that the calculated values are comparable, we also calculated the difference between habitats located on different devices (and thus inoculated with different cultures) for the type-5 devices. Here we find a median difference of *d*_*different*_ = 0.38 (25%-75% quartiles = 0.37-0.39) which is similar to that of the type-1 and 2 devices (*d*_*different*_ = 0.38 median, 25%-75% quartiles = 0.37-0.40; *p* = 0.9, Wilcoxon rank sum test), indicating that the calculated values for the differences between population distributions are comparable between the type-5 and the type-1 and 2 devices.

The results of the type-4 and 5 devices (Additional files 9C, 10 and 12) show that neighboring habitats do not become similar when they are inoculated from different cultures, while habitats inoculated from the same cultures remain similar even when they are separated in space and invaded in reverse orientation. This strongly suggests the higher degree of similarity in population distributions between habitats on the same device, colonized by the same culture sets, as observed in the type-1 and 2 devices (Figure 6 and Additional files 2 and 3) is not a consequence of abiotic factors or other extrinsic variation, but rather that it is caused by an underlying biological mechanism intrinsic to the colonizing populations.

We hypothesized that the similarity between replicate habitats was a consequence of inoculating them with the same initial cultures. However, when we compare the two habitats on type-5 devices that were inoculated from the same culture set we found that the difference between population distribution in habitats inoculated from the same culture set (*d*_*same*_ = 0.35, median, 25%-75% quartiles = 0.28-0.37) is not significantly different from the difference between habitats inoculated from different culture sets (but still located on the same device, *d*_*different*_ = 0.32, median, 25%-75% quartiles = 0.27-0.42, *p* = 0.74, Wilcoxon signed rank test, N = 8, Additional file 9C).

Which mechanisms are instead causing the observed similarity in population distributions between the replicate habitats in device types-1 and 2 is currently unclear. Nevertheless, our results do suggest that colonization patterns are strongly affected by some (currently unknown) deterministic factors, while stochastic effects during the colonization process have only a limited influence.

## Discussion

We consistently observe colonization waves entering the habitat from both ends with wave profiles and velocities (Additional file 5) comparable to those reported for population waves in previous studies [29,30,33,43]. This indicates that the qualitative features of bacterial colonization waves are robust to changes in habitat geometry and suggests that our results could be of importance in natural habitats with complex spatial structure ranging from the micrometer to the millimeter scale. Our habitats are typically colonized by two waves of low cell density (labeled α and β in Figure 1D) followed by a single high-density wave (labeled γ in Figure 1D). This succession of multiple waves is reminiscent of the observations by Adler, who showed that multiple waves can form both in capillary tubes and on agar plates, where each wave consumes a different set of nutrients [2,6]. We further studied the local interaction between colliding waves and observed, similar to previous work on agar plates, that when waves collide (Figures 2 and 3) they can either reflect back, continue in the same direction with an altered velocity (“refract”), or collapse to form a distinct and localized sessile population [2,19,38-41]. Moreover by using differentially labeled cells we have shown that although mixing does occur to some degree, the bacteria traveling within a wave remain mostly together after colliding with another wave. We observed two main differences in relation to earlier experiments: (i) previously [19], waves have been observed to either reflect, refract or collapse (depending on the agar concentration, pH and strains used) but not to split into simultaneous combinations of these options. We observe that all three outcomes are simultaneously possible at a single collision, although there is a large variation between experiments in the distribution of the incoming wave over these components (Figure 3); (ii) previously [38], it has been observed that a localized population (formed after a collision) can emit a reflected wave after about one hour (a timescale which has been argued to be required by the cells to switch to a different nutrient). In contrast, the reflected waves observed in our devices reverse direction within 10 minutes, without first forming an observable stationary population.

Driven by the results described above we designed a third type of device (type-3; Figure 5A) with which we demonstrated that traveling populations confined to separate, but chemically coupled, habitats still influence each others colonization dynamics and exhibit “collisions”, despite having exclusive access to vacant patches (Figure 5). This shows that chemical interactions are the main mechanisms underlying the collision patterns of colonization waves as well as of expansion fronts. These interactions could possibly be mediated by small diffusible molecules. Using a typical diffusion constant of D = 5·10^−6^ cm^2^/s for such molecules, we find that diffusion between the two coupled habitats takes place on the order of 0.1 s, while the diffusional range at the time-scales probed in this study (i.e. 10 min) is on the order of 1 mm (i.e. 7 patches). Therefore diffusible molecules could indeed be involved in the observed interactions of population waves and in the short-range interactions between population fronts. The long distance interactions (over ~1 cm, Figure 4E,F) however, happen at time scales much faster (~1 h) than those of diffusion (~15 h). These interactions might therefore be mediated by different mechanisms. Nevertheless, it is likely that at least the short range (*d* ~ 1 mm) interactions are caused by some form of habitat conditioning (e.g. consumption of nutrients, excretion of metabolites, chemoattractants and/or repellents) and/or by cell-signaling.

It is interesting to note that when two strains are co-cultured together before inoculation, they colonize a habitat together and form a mixed metapopulation (Figure 4G and Additional file 7). In contrast, if the strains are cultured independently and invade the habitat from opposite ends, they form two distinct and competing metapopulations that do not mix when they meet in the habitats (Figure 4). Furthermore, we have demonstrated that population waves and expansion fronts from opposite sides remain spatially segregated, even if they originate from the same culture (Additional file 4B-D) and consist of cells of the same strain.

These observations, together with the observed interactions of colonization waves and expansion fronts, suggest that the spatial segregation of different (sub)populations is caused by some sort of avoidance mechanism. Observations in other microbial species could hint at possible mechanisms for such avoidance between different populations. For example, in *Bacillus subtilis* and *Paenibacillus dendritiformis* chemo-repellents have been suggested to cause self-avoidance of colony branches [45,46]. In *P. dendritiformis* the excretion of a growth inhibiting lethal factor causes the formation of a well defined boundary between sibling populations [47,48]. A genetic system for self- versus non-self recognition was found to mediate boundary formation between different *Proteus mirabilis* strains [49] and in *Dictyostelium discoideum* the cell cycle phase and nutritional status of subpopulations has been shown to affect their relative contribution to spore and stalk cell populations [50]. However, to the best of our knowledge, such mechanisms have not (yet) been shown to be of importance in *E. coli*. Furthermore, it would be interesting to see if the current models of population waves [29,30,43,51,52] are capable of producing the local collision patterns on the timescales we observed in our experiments.

In the type-1 and 2 devices we observed a remarkable similarity between colonization patterns in replicate habitats on the same device. Population distributions in habitats on the same device, which were inoculated from the same set of initial cultures, are significantly more similar to each other (as measured by the Euclidian distance between occupancy patterns) than to the patterns in habitats on different devices which were inoculated from different culture sets (Figure 6, Additional files 2 and 3). Using a device of type-4 we showed that population distributions in habitats inoculated from the same cultures are similar even when the habitats are not parallel to each other (Additional file 10), while using devices of type-5 we showed that population distributions in habitats inoculated with different cultures do not become similar when the habitats are located next to each other on the same device (Additional files 9C and 12). Together these data strongly suggest that the observed similarity between replicate habitats in type-1 and 2 devices is not an artifact of our experimental design, but is rather caused by a biological mechanism.

All devices were prepared by strictly adhering to the experimental protocol (see Methods); therefore, we suspected that the variation in colonization patterns between different devices was caused by differences in the initial cultures used to inoculate the habitats. We tested this hypothesis using the type-5 devices, however, for these devices we found that habitats located on the same device and inoculated from the same set of initial cultures were not more similar to each other than to habitats located on the same device but inoculated from different culture sets (Additional files 9C and 12). We therefore have no conclusive evidence that the degree of similarity between habitats is caused by the initial cultures used to inoculate them, however, our results suggest that the initial cultures might affect colonization patterns to some degree. At the moments it is unclear which other mechanism causes the observed similarity between the replicate habitats in the type-1 and 2 devices.

It should be noted that the actual habitats in all device types are identical and that the only differences are in the number of parallel habitats, the inlets and the inoculation procedure (see Methods). Therefore, the only two differences between type-1 and 2 devices and type 5 devices are: (i) the reduced number of replicate-habitats (2 instead of 5). Additional file 2 shows that in some cases there is substantial variation between the population distributions in replicate habitats on the same device (e.g. devices 5 and 6, Additional file 2). Therefore, having only two replicate habitats could reduce the likelihood of detecting a significant effect of the initial culture on the similarity in population distributions; (ii) in type-5 devices habitats inoculated from the same cultures are further apart (900 μm compared to 300 μm) and are separated by a habitat inoculated from a different culture set; and (iii) for the type-5 devices variation in the preparation of overnight cultures was reduced: instead of taking a sample (of undefined volume) of the frozen −80°C stock, a defined volume of a thawed aliquot of this stock was used to start the overnight cultures (see Methods).

Our results show that spatial proximity is not sufficient to make patterns of different cultures similar (device type-5), nor is it required to keep patterns of the same cultures similar (device type-4). Nevertheless, we cannot rule out that there is some limited coupling between the habitats. There is a possibility that weak coupling works in concert with culture history to produce similar patterns, but is not sufficient to produce an effect on its own if neighboring populations do not originate from the same initial cultures.

Nevertheless, we do observe a striking and significant degree of similarity between neighboring habitats located on the same device and inoculated from the same initial cultures (Figure 6, Additional files 2 and 3) that to the best of our knowledge cannot be explained by any abiotic factors. Despite the many open questions, our results do show that colonization patterns are in a large part shaped by (currently unknown) deterministic factors, while stochastic effects are only of limited importance.

## Conclusion

We studied the invasion and colonization of spatially structured habitats by two neutrally labeled strains of *E. coli*. One-dimensional arrays of habitat patches were colonized by a succession of colonization waves followed by an expansion front. The interactions between the two invading populations lead to complex, but reproducible, spatiotemporal patterns which are dominated by the collisions of colonization waves and expansion fronts. Colliding colonization waves each split into a combination of a stationary population, a reflected wave, and a refracted wave; while expansion fronts entering from opposite sides remain spatially segregated and compete for habitat space. As these interactions also occur when the two populations are in separate, but diffusionally coupled habitats, we can conclude that interactions between (sub)populations are mediated by chemical fields and do not require physical contact.

Finally, we showed that the outcome of the colonization process is influenced by a culture’s history, as the relative doubling time of the initial cultures in bulk conditions correlates with the relative occupancies obtained in the habitats. Together, our data show the important roles of chemical coupling between populations and culture history in determining the colonization of spatially structured habitats.

## Methods

### Strains

Experiments were performed with two fluorescently labeled strains of wild type *Escherichia coli:* JEK1036 (W3110 [*lacZY::GFPmut2*], green) and JEK1037 (W3110 [*lacZY::mRFP1*], red). These strains are isogenic except for the fluorescent markers inserted in the lac operon [42]. Furthermore, we used the non-chemotactic, smooth-swimming strain JEK1038 (W3110 [*lacZY::GFPmut2, cheY::frt*], green) which was derived from strain JEK1036 by *cheY* deletion. Fluorescence expression was induced by adding 1 mM of Isopropyl β-D-1-thiogalactopyranoside (IPTG, Promega) to the culture medium.

### Growth conditions, the initial culture, and the inlet hole populations

We use the term *initial culture* to refer to the specific batch culture used to inoculate a habitat. Different initial cultures of the same strain all originate from the same −80°C glycerol-stock, but have been grown independently following the protocol described below.

Overnight cultures were grown in a shaker incubator for approximately 17 hours at 30°C in 3 ml Lysogeny Broth medium (LB Broth EZMix, Sigma-Aldrich). Cultures were subsequently diluted 1:1000 in 3 ml LB medium supplemented with 1 mM IPTG and grown for another 3.5 hours before inoculating the microfabricated devices.

For devices of types 1 to 4 overnight cultures were started by transferring a sample of the frozen stock to a culture tube using a sterile pipet tip. After 1000× back dilution the cultures were grown for 210 ± 21 min (mean ± sd) to an optical density at 600 nm (OD_600_) of 0.20 ± 0.07 (mean ± sd).

For experiments performed with mixed initial culture of strains JEK1036 and JEK1037, the two strains were grown overnight independently and mixed in 1:1 ratio during back dilution (volume ratios were determined using the OD_600_ of the overnight cultures). The mixed culture was subsequently grown for 203 ± 11 min (mean ± sd) before inoculation of the device at a combined OD_600_ of 0.17.

For devices of type 5 the original −80°C glycerol-stock was split into aliquots, overnight cultures were started by adding 6 uL from a thawed aliquot to a culture tube and were subsequently grown for 17 hours ± 3 min. After 1000× back dilution the cultures were grown for 210 ± 2 min (mean ± sd) to an OD_600_ of 0.34 ± 0.04 (mean ± sd). All initial cultures (of a given strain) used in the same experiment were started from the same −80°C aliquot.

### Imaging and data processing

Time-lapse fluorescence imaging of the bacterial populations was done using computer controlled microscopes. Three microscope setups were used: (i) an Olympus IX81 motorized inverted microscope controlled with the MicroManager 1.4.6 software [53], equipped with a 10× 0.25NA objective and Hamamatsu ORCA-R2 camera; (ii) a Nikon Eclipse Ti+E inverted microscope controlled with the Nikon Elements AR software, equipped with a 10× 0.45NA objective and an Andor iXon 885 emCCD camera; and (3) an Olympus IX81 motorized inverted microscope controlled with the MicroManager 1.4.14 software [53], equipped with a 20× 0.75NA objective and Andor Neo sCMOS camera. Devices were scanned every 10 minutes for at least 20 hours. Fluorescence images were cropped, concatenated and rescaled using the software ImageJ 1.45 [54]. Further analysis of the data was done using Matlab 2011b and statistical analysis was done using R 1.15.1 for Mac [55] and Matlab 2013a.

### Microfabricated devices

Devices were fabricated from silicon as described in Keymer et al. [34] using either a one-step (device types 1,2,4 and 5) or two-step (device type 3) process of photolithography and reactive ion etching. Inlet holes were hand drilled using a sandblaster and have a volume of approximately 200–500 nl (mean ± sd = 311 ± 65 nl, volumes estimated for 44 inlet holes on 6 devices by assuming a truncated-cylinder shape where the depth (=550 μm) is given by the thickness of the silicon wafer and the dimensions of the top and bottom surfaces were estimated from images taken with a stereo-microscope). Devices were sealed with a polydimethylsiloxane (PDMS, SYLGARD 184) covered glass coverslips. Devices were used only once.

Bacteria grow in 100 × 100 × 5 μm^3^*habitat-patches* (*patch* for short, Figure 1C); habitat-patches are connected to form *habitats*, which consist of a linear array of 85 patches coupled by connectors of 50 × 5 × 5 μm^3^ (Figure 1C). Each *microfabricated device* (*device* for short, Figure 1A-B) consists of multiple habitats etched in the same piece of silicon and sealed with a common coverslip (see below). Habitats are connected to *inlet holes* using *inlet channels* (Figure 1A-B).

Five types of microfabricated devices were used, in all cases the actual habitats are the same, however devices differ in the number of parallel habitats, the arrangement of the inlets and the inoculation procedure.

#### Type 1

Each device consists of five independent habitats (Figure 1A). Each habitat is connected on both sides to separate inlet holes by 3.1 mm long, 5 μm wide and 5 μm deep inlet channels (Figure 1A). Habitats are separated by 200 μm of solid silicon and are sealed on the top with a PDMS layer, ensuring that there is no liquid connection between different habitats.

#### Type 2

Each device consists of five habitats sharing a single inlet (Figure 1B). A 25 μm wide, 2.6 mm long and 5 μm deep inlet channel branches in five 5 μm wide, 9 mm long and 5 μm deep channels which connect all five habitats to a single inlet hole (Figure 1B). Except for the shared inlet there is no liquid connection between the five habitats.

#### Type 3

Each device consists of two independent sets of two diffusionally coupled habitats (Figure 5A). Each set consists of two habitats (i.e. top and bottom habitat) separated by 15 μm that are coupled by 200 nm deep nanoslits of 15 × 15 μm^2^ that are spaced 5 μm apart (Figure 5A). These nanoslits allow for the diffusion of chemicals but are too thin for cells to swim through [44], thereby confining cells to a single habitat. The top and bottom habitats are both connected to independent inlet holes by 5 μm wide, 3.5 mm long and 5 μm deep inlet channels.

#### Type 4

Identical to type 1, except that only the outer two habitats are used (Additional file 10B). The three inner habitats are completely sealed off, creating a separation of 1.2 mm between the two habitats.

#### Type 5

Identical to type 1, except that the central habitat (habitat 3) is sealed off.

### Device preparation and imaging conditions

Microfabricated devices were filled with LB medium containing 1 mM IPTG. Habitats were inoculated by pipetting 3 μl of initial culture onto an inlet hole. Excess medium was let to evaporate and the inlet holes were subsequently sealed with PDMS. Lastly, a glass coverslip was applied to cover the back of the device. Inlet holes are inoculated with approximately 10^5^ cells (assuming that cells from the excess medium do not enter the inlet hole). The devices were imaged at 26°C.

The culture medium is not refreshed after sealing the device; therefore the use of a rich medium is required to sustain a sufficient increase in population size. We still observe cells swimming through the habitats four days after inoculation. Furthermore, the location of the boundary between the two populations fronts shifts over time. Together this strongly suggests that nutrients are not fully depleted after the initial colonization of the device and that most of the fluorescence signal observed during the first 18 h originates from living cells.

### Experimental scheme

The experimental scheme for the main datasets is summarized in Additional file 11.

**Type-1 devices** (6 devices, 24 habitats): On each day a single device was imaged; all habitats on the same device were inoculated from a single set of initial cultures (Devices 1–6, Additional file 11). The kymographs of all successfully invaded habitats are shown in Additional file 2.

**Type-2 devices** (5 devices, 24 habitats): In all but one case a single device was imaged per day (Devices 7–9, Additional file 11); in the remaining case 2 independent devices, both inoculated from the same set of initial cultures, were imaged in parallel on the same microscope setup (Devices 10 and 11, Additional file 11). In all cases all habitats on the same device were inoculated from a single set of initial cultures. The kymographs of all successfully invaded habitats are shown in Additional file 3.

**Type-3 devices** (2 devices, 3 sets of coupled habitats): The two sets of diffusionally coupled habitats on the same device were prepared identically by inoculating the upper habitat from the left and the lower habitat from the right from the same initial culture of strain JEK1036 (Figure 5A). The kymographs of all successfully invaded habitats are shown in Figure 5 and Additional file 8.

**Type-4 device** (1 device, 2 habitats): The two habitats were inoculated from the same cultures set, but in reverse orientation (i.e. red from the left in habitat 1 and from the right in habitat 2, Additional file 10B). The kymographs of all successfully invaded habitats are shown in Additional file 10.

**Type-5 devices** (8 devices, 14 habitats): Each device was inoculated from two independent overnight cultures which were started from the same −80°C aliquot and grow next to each other in the incubator. Each culture set was inoculated in two habitats, in such a way that neighboring habitats contained different culture sets (i.e. culture set 1 in habitats 1 & 3 and culture set 2 in habitats 2 & 4, Additional file 11). The kymographs of all successfully invaded habitats are shown in Additional file 12.

**Control experiments:** (i) non-chemotactic strain (3 type-1 devices), see Additional file 4A and the accompanying data set [56]; (ii) red-green co-culture (1 type-1 and 1-type 2 device), see Figure 4G and Additional file 7; (iii) same initial culture from both sides (1 type-1 device using JEK1036, see Additional file 4B-D; 2 type-5 devices where habitats 1&2 were inoculated on both sides with JEK1036 and habitats 4&5 with JEK1037, see accompanying data set [56]).

### Estimating population densities by calculating patch occupancy

We monitored the bacterial metapopulations using their fluorescence emission. However, the fluorescence intensity per cell is different for the two fluorescent proteins and changes with growth phase, making it an imprecise measure of population density. Instead, we estimated population densities by measuring the area fraction of the patches occupied by bacteria, i.e. the occupancy. A pixel in each color channel of an image is considered to be occupied by bacteria if its intensity is above a dynamically calculated threshold. Thresholds are calculated using a previously published [35] custom-build Matlab-algorithm which fits a Gaussian distribution to the auto-fluorescence intensity of the culture medium and sets the threshold (within predefined bounds) to a value of 3 to 5 standard deviations above the background intensity. Subsequently, the images are converted to binary images using this threshold and the occupancy value, which ranges from 0 (strain absent from patch) to 1 (strain fully covering patch), is calculated for each color-channel. The result of this procedure can be seen in Figure 2: Figure 2B shows the acquired fluorescence image, while Figure 2A shows the calculated occupancy values for the red channel (top) and green channel (bottom, see also Figure 3). It should be noted that the occupancy is not a linear measure of population density, as it cannot distinguish between mono- and multilayers of cells, causing it to saturate at high bacterial densities. Furthermore, the green channel has typically a higher background fluorescence intensity compared to the red channel, this can lead to differences in the detection of faint or motion blurred cells between the two channels. Nevertheless, we believe that occupancy is a more reliable estimate of population density than fluorescence intensity due to its relative insensitivity to differences in the per-cell fluorescence intensity between fluorescent proteins and with growth phase.

### Quantitative similarity measure between spatiotemporal patterns of occupancy

To estimate the degree of similarity between cell distributions in two habitats, the Euclidean distance between their occupancy kymographs is calculated. Each pixel in the occupancy kymographs represents a vector [*r(t,k);g(t,k)*] of the occupancies of the green strain (JEK1036, *g(t,k)*) and red strain (JEK1037, *r(t,k)*) for a given patch *(k)* at a given time *(t)*. The difference *(d)* between kymographs is calculated by taking, for each pixel and color channel, the square of the difference in occupancies between the two habitats and summing this over all pixels:

(1) $$d=\sqrt{\frac{1}{2M}\cdot \sum_{t}\sum_{k}\left({\left[r_{1}\left(t,k\right)-r_{2}\left(t,k\right)\right]}^{2}+{\left[g_{1}\left(t,k\right)-g_{2}\left(t,k\right)\right]}^{2}\right)},\;0\leq d\leq 1$$

where *r*_*1*_*(t,k)* and *r*_*2*_*(t,k)* are the occupancies of strain JEK1037 in patch *k* at time *t* obtained for habitats 1 and 2 respectively. Similarly, *g*_*1*_*(t,k)* and *g*_*2*_*(t,k)* are the occupancies of strain JEK1036 in patch *k* at time *t* calculated for habitats 1 and 2 respectively. The factor 2 *M* (where *M* is the total number of pixels in the kymograph) normalizes *d*, such that it ranges from 0 for identical patterns to 1 for maximally different patterns. The difference is calculated over the period between 3 and 18 hours after inoculation. The first 3 hours are excluded as this time is required to setup the image acquisition and the end limit of 18 hours is chosen as most patterns have stabilized by this time (Additional files 2, 3 and 12). It should be noted that the Euclidean distance between two patterns is mostly affected by differences in high-density regions occupying large expanses (in space and/or time, *e.g.*, the expansion fronts), it is therefore hardly affected by more subtle aspects of the colonization pattern (*e.g.*, the colonization waves).

### Statistical analysis

To compare two datasets we used the Student’s t-test where possible; when variances were unequal (as determined using a two-sample equal variance test) the unequal variances (Welch’s) t-test was used; for datasets that were not normally distributed (as judged by visual inspection) the Wilcoxon rank-sum (i.e. Mann–Whitney U) or the Wilcoxon signed rank test (for paired samples) was used.

The similarity between cell distributions in different habitats was assessed by calculating for each habitat the average difference to habitats inoculated from the same set of initial cultures (*<d*_*same*_*>*) and the average difference to habitats inoculated from different sets of initial cultures (*<d*_*different*_*>*). For devices of types-1 and 2 these differences were calculated using habitats on all devices of a given type, while for devices of type-5 comparisons were only made between habitats located on the same device.

To test whether there is a significant difference between *<d*_*same*_*>* and *<d*_*different*_*>* for the devices of types 1 and 2 we used a randomization test. To get a single observable per habitat, the ratio of these two differences was taken: *d*_*relative*_ = < *d*_*same*_ >/< *d*_*different*_ >, when *d*_*relative*_ is smaller than 1 patterns are less different when they are inoculated from the same set of cultures. The difference between spatiotemporal patterns is a comparative measure; the ratio *d*_*relative*_ of a given habitat therefore depends on the patterns in all other habitats. To deal with this dependence between data points we assessed significance using a randomization test, where we randomize with respect to the set of initial cultures. For each device type (type 1 and 2) we calculated the average of the log transformed *d*_*relative*_ (*<*log[*d*_*relative*_]>*)* by averaging over all habitats, we then recalculated this measure after randomizing the spatiotemporal patterns by assigning each observed spatiotemporal pattern to a randomly chosen habitat. The randomizations were performed 10.000 times and *p*-values were calculated by taking the fraction of cases where *<*log[*d*_*relative*_]> after randomization was smaller than the *<*log[*d*_*relative*_]> of the original, non-randomized, data set. Two devices of type 2 were both inoculated from the same set of initial cultures (Devices 10 and 11, Additional files 3 and 11), for this analysis the habitats on these devices were grouped together.

### Strain neutrality

Neutrality of the strains during bulk growth has been previously described [42] and was confirmed here by measuring the average doubling time of cultures during the 3.5 hours of growth before inoculation of the devices. There was no significant difference in the average doubling time of strains JEK1036 (green) and JEK1037 (red, mean ± sd = 35.5 ± 2.0 min and 36.0 ± 2.6 min respectively, paired Student’s t-test, *p* = 0.06, N = 23). Growth curves for the two strains in bulk conditions are shown in Additional file 1.

To test for marker neutrality during growth in the microfabricated devices, we compared the occupancies of the two strains in the habitats. We determined the habitat-wide average occupancies by averaging the occupancy over all patches in a habitat. Population distributions in habitats inoculated from the same culture set are not independent from each other, therefore we average over all habitats inoculated from the same culture set. Additional file 6D shows the resulting average occupancy as function of time. When comparing the average occupancy at the end of the experiment (*t* = 18 h), we do not detect a significant difference between the two strains (occupancy = 0.28 (0.14-0.33) for JEK1036 and 0.35 (0.17-0.41) for JEK1037 (median, (25%-75%) quantiles), (paired) Wilcoxon signed rank test, *p* = 0.29, N = 26, Additional file 6F). However, when comparing the occupancy averaged over the entire colonization process (3 < *t* < 18 h), we observe a slightly higher occupancy for the red cells (occupancy = 0.22 (0.14-0.31) for JEK1036 and 0.26 (0.21-0.43) for JEK1037 (median, (25%-75%) quantiles), (paired) Wilcoxon signed rank test, *p* = 0.046, N = 26, Additional file 6F). Despite this difference in the average occupancy obtained in the habitats, both strains are able to reach a majority in a habitat. In Additional file 6E it can be seen that in 9 out of 26 experiments strain JEK1036 (green) occupies the majority of the habitats (*p* = 0.17, sign-test, N = 26), while in 6 experiments strain JEK1036 obtains a two-third majority (compared to 9 experiments for JEK1037). These last results suggest that the two strains are neutral, even tough strain JEK1037 does appear to obtain higher average occupancies in the habitat. It should be noted that the occupancy is not a direct measure for population densities (as discussed previously). Therefore we performed control experiments where we inoculated habitats with a 1:1 mixture of the two strains. Here we observed that the two strains remain fully mixed (Figure 4G, Additional file 7). Furthermore, we observed that both strains are able to drive the other strain almost completely out of the habitat (e.g. compare device 2, Additional file 2 with device 11, Additional file 3). These last two results, together with the isogenic background of the strains, suggest that the two strains are on average neutral when colonizing the habitats.

### Wave velocity

Wave velocities were determined manually by fitting a line on waves visible in kymographs of the average fluorescence intensity per patch. If a wave changed velocity it was piecewise fitted using either two or three linear segments, for further analysis only the velocity just after entering the habitat was used. Waves were manually classified as either α, β or γ waves. In all experiments a maximum of two low intensity waves were observed, which were classified as α and β waves (for the first and second wave respectively). The high intensity wave at the leading edge of the expansion front was classified as a γ wave, even if the α and/or β waves were not visible. There is no significant difference between the wave velocities of strains JEK1036 (green) and JEK1037 (*v* = 46 μm/min (median), *N* = 126 and *v* = 48 μm/min, *N* = 86, respectively, Wilcoxon rank sum test, *p* = 0.25) and for all further analysis the wave velocities of both strains were combined.

## Availability of supporting data

The data sets supporting the results of this article are available in the 3TU.Datacentrum repository [56], [doi:10.4121/uuid:f5603abf-bf15-4732-84c0-a413ce7d12d3], [http://dx.doi.org/10.4121/uuid:f5603abf-bf15-4732-84c0-a413ce7d12d3].

## Competing interests

The authors declare that they have no competing interests.

## Authors’ contributions

SvV participated in conceiving the study, participated in its design, performed the experiments and data analysis and drafted the manuscript. FJHH contributed data analysis tools, helped to perform experiments, and helped to draft the manuscript. TW performed exploratory experiments. PG performed exploratory experiments and participated in the design of the study. JEK conceived of the study, participated in its design and coordination and participated in drafting the manuscript. All authors read and approved the final manuscript.

## Supplementary Material

Additional file 1 **Growth curves of strains JEK1036 and JEK1037 in bulk conditions.** Growth curves are shown for strains JEK1036 (in green) and JEK1037 (in red), for each strain 3 independent cultures were grown in 200 ml LB in 500 ml flasks at 30°C. For each sample the OD_600_ was measured in triplicate and their average value was used. Error bars indicate sem. The inset shows the growth curve using linear y-scale for the first 15 hours. Click here for file

Additional file 2 **Overview of all devices with separate inlets (type 1).** (A) Each kymograph shows the average occupancy per patch in a single habitat. Kymographs for the five parallel habitats in a single device are shown next to each other. Note that all habitats on the same device are inoculated from the same culture set. (B) The device-wide averages of the occupancies of strains JEK1037 (*R* red) and JEK1036 (*G* green) and the red fraction (*f*_*r*_ black) are shown as function of time. Dashed lines indicate mean ± sem. The red fraction (*f*_*r*_) is calculated for each habitat as *f*_*r*_ = *r*/(*r* + *g*)*,* where *r* and *g* are the habitat-wide average occupancies of strains JEK1037 (red) and JEK1036 (green) respectively. Habitats where one (or both) of the strains failed to enter (e.g. when there is a constriction in one of the inlet channels) were excluded from the analysis and are shown as grey panels in this figure. Click here for file

Additional file 3 **Overview of all devices with a single inlet (type 2).** (A) Each kymograph shows the average occupancy per patch in a single habitat. Kymographs for the five parallel habitats in a single device are shown next to each other. Note that all habitats on the same device are inoculated from the same culture set. (B) The device-wide averages of the occupancies of strains JEK1037 (*R*, red) and JEK1036 (*G*, green) and the red fraction (*f*_*r*_ black) are shown as function of time. Dashed lines indicate *mean* ± *sem*. The red fraction (*f*_*r*_) is calculated for each habitat as *f*_*r*_ = *r*/(*r* + *g*), where *r* and *g* are the habitat-wide average occupancies of strains JEK1037 (red) and JEK1036 (green) respectively. Habitats where one (or both) of the strains failed to enter (e.g. when there is a constriction in one of the inlet channels) were excluded from the analysis and are shown as grey panels in this figure. Note that devices 10 and 11 were inoculated from the same initial cultures. Click here for file

Additional file 4 **Interactions between populations originating from the same initial culture.** (A) Kymograph of fluorescence intensity for a type-1 device inoculated at both sides with the non-chemotactic, smooth-swimming, strain JEK1038 (Δ*cheY*). (B) Kymograph of fluorescence intensity for one habitat in a type-1 device that was inoculated at both sides with cells coming from the same initial culture of strain JEK1036. (C) Enlarged part of panel B. (D) Enlarged part of a different habitat in the same device as shown in panels B and C. Click here for file

Additional file 5 **Bacterial colonization waves in patchy habitats.** (A) Wave profile of the α wave shown in Figure 1D, shown here as the area fraction occupied per patch (occupancy) as function of space, different lines show the profile for *t* = 210 min to *t* = 250 min in steps of 10 minutes. (B) Wave profile for the β wave shown in Figure 1D, different lines show the profile for *t* = 320 min to *t* = 350 min in steps of 10 minutes. (C) Wave profile for the γ wave and expansion front (F) shown in Figure 1D, different lines show the profile for *t* = 390 min to *t* = 430 min in steps of 20 minutes. (D) Distribution of wave velocities (of strains JEK1036 and JEK1037 combined) for α (red), β (green) and γ (blue) waves. Click here for file

Additional file 6 **Effects of the strain and the bulk growth parameters on the occupancy obtained in the habitats.** (A-C) Relation between the occupancy obtained in the habitat and three bulk growth parameters: (i) *OD overnight*: the OD_600_ of the overnight culture; (ii) *OD start*: OD_600_ of the initial culture (iii): *t*_*d*_: the average doubling time of the initial culture during growth after back-dilution. Relative values are calculated for each culture-set by dividing the measurement for strain JEK1036 (green) by the corresponding measurement for strain JEK1037 (red) and taking the log of this ratio, i.e. as log[X(green)/X(red)], where X represents the measure of interest (A) Relation between bulk growth parameters and the occupancy at *t* = 18 h, for strain JEK1036 (green diamonds) and strain JEK1037 (red circles). (B) Relation between the relative occupancy averaged over the entire colonization process (i.e. 3 < *t* < 18 h) and the relative bulk growth parameters. (C) Relation between the relative occupancy at *t* = 18 h and the relative bulk growth parameters. Linear regression lines are shown in red, r^2^ values (of Pearson correlation) and the corresponding *p*-values are shown above each panel. (D-F) Comparison of the occupancy obtained in the habitat by strain JEK1036 (green) and JEK1037 (red). The data shown is based on all habitats of devices of types-1, 2 and 5. Measurements of habitats inoculated from the same culture set were averaged before combining them with data from other experiments. (D) Average occupancies of strains JEK1036 (green solid line) and strain JEK1037 (red solid line) as function of time, dashed lines indicate 95% confidence intervals. (E) Occupancy of strain JEK1036 plotted as function of the occupancy of strain JEK1037 at *t* = 18 h. Each point corresponds to the average occupancy obtained in the habitats inoculated from the same culture set. Symbols indicate the device type: plus-signs (+): type-1, stars (*): type-2, crosses (x): type-5. (F) Distribution of occupancies of strain JEK1036 (G) and JEK1037 (R) at the end of the colonization (*t* = 18 h) and averaged over the entire colonization phase (3 < *t* < 18 h). Click here for file

Additional file 7 **Devices inoculated at both ends with a mixed culture of strains JEK1036 and JEK1037.** (A) Kymographs of fluorescence intensity for a device with separate inlets (type 1; Figure 1A) inoculated at both ends with a single mixed culture of strains JEK1036 and JEK1037, with the kymograph of RFP (JEK1037) on the left, of GFP (JEK1036) in the middle and of the combined colors on the right. Note how the two strains remain mixed throughout the experiments, in contrast, the strains remain spatially segregated when inoculated from opposite sides of the habitat, as shown in panel D. (B) Kymographs of fluorescence intensity for a device with a single inlet (type 2; Figure 1B) inoculated at both ends with a single mixed culture of strains JEK1036 and JEK1037, with the kymograph of RFP (JEK1037) on the left, of GFP (JEK1036) in the middle and of the combined colors on the right. (C) Kymographs of fluorescence intensity for a different habitat in the same device as shown in panel B, inoculated at both ends with a single mixed culture of strains JEK1036 and JEK1037, note the similarity between the patterns in panels B and C. (D) As reference the kymographs are shown for the habitat shown in Figure 4A, with the kymograph of RFP (JEK1037) on the left, of GFP (JEK1036) in the middle and of the combined colors on the right. Click here for file

Additional file 8 **Interactions between chemically coupled, but physically separated population.** Kymographs are shown for two type-3 devices. The fluorescence intensities of the top and bottom habitat are superimposed: cells in the top habitat are shown in red and cells in the bottom habitat in green. Note that both habitats are inoculated from the same (JEK1036, green) culture, and that the bacteria in the upper and lower habitats are spatially confined to their own habitat. Click here for file

Additional file 9 **Similarity between spatiotemporal patterns.** Panels (A and B) show the results of a randomization test performed to assess the effect of the initial cultures on the degree of similarity between population distributions. For each habitat we calculated the ratio of the average difference in population distributions of habitats inoculated from the same cultures (*<d*_*same*_*>*) relative to the average difference to all habitats inoculated from different cultures (*<d*_*different*_*>*): *d*_*relative*_=*<d*_*same*_*>*/*<d*_*different*_*>*. The red arrows indicate <*d*_*relative*_>, obtained by averaging log[*d*_*relative*_ ] over all habitats of a given device type. The blue distribution shows the values of *<d*_*relative*_*>* obtained using 10.000 randomizations, where each population distribution was assigned to a randomly chosen habitat. Note that values of *d*_*relative*_ were log transformed before averaging, the figure shows the back-transformed values. (A) Devices of type-1. (B) Devices of type 2. Note how in all cases the *<d*_*relative*_*>* for the real dataset (in red) is much lower than the *<d*_*relative*_*>* obtained from the randomized dataset (in blue). *** indicates *p < 0.001*. (C) Comparison of the degree of similarity observed in type-1 and 2 devices combined to that observed in devices of type-5. For both groups the differences between population distributions in habitats inoculated from the same culture set (*d*_*same*_) and the difference between population distributions in habitats inoculated from different culture sets (*d*_*different*_) is shown. Values of *d*_*same*_ and *d*_*different*_ obtained for habitats inoculated from the same culture sets were averaged together. N.S. indicates *p* > 0.05 in a Wilcoxon rank sum test (comparison of *d*_*different*_ between type 1 and 5 devices) or Wilcoxon signed rank test (comparison between *d*_*same*_ and *d*_*different*_ for type 5 devices). Click here for file

Additional file 10 **Device type-4 where the two habitats where inoculated in reverse orientation.** (A) Kymograph of fluorescence intensity for a device of type-4, where only the two outer most habitats are used. The orientation of inoculation was reversed for the two habitats, i.e. the red strain was inoculated from the right into habitat 1 and from left into habitat 2, see panel B. Note that the kymograph of habitat 2 is horizontally mirrored to reveal the similarity with habitat 1. (B) Schematic of the inoculation locations. Click here for file

Additional file 11 **Experimental Protocol.** Protocol for the experiments using type-1 (top part), type-2 (middle part) and type-5 (lower apart) devices. Devices 10 and 11 (type-2) were imaged in parallel on the same microscope setup, after being inoculate from the same set of initial cultures. For devices of types 1 and 2 overnight cultures were started by taking a sample (of undefined volume) from a single −80°C stock for each strain, for devices of type-5 these same −80°C stocks (one for each strain) were split into aliquots and each overnight culture was started using a defined volume of a thawed aliquot. The following morning cultures were back-diluted 1:1000 to result in the initial culture with which the devices were inoculated. Click here for file

Additional file 12 **Overview of all devices of type-5.** Each kymograph shows the average occupancy per patch in a single habitat. Kymographs for the four parallel habitats in a single device are shown below each other. Note that devices were inoculated from two different sets of initial cultures: habitats 1 and 3 from culture set 1 and habitats 2 and 4 from culture set 2. Habitats where one (or both) of the strains failed to enter (e.g. when there is a constriction in one of the inlet channels) were excluded from the analysis and are shown as grey panels in this figure. Click here for file
