# Supplementary figures and images for: The effects of chemical interactions and culture history on the colonization of structured habitats by competing bacterial populations
Source: BMC Microbiol. 2014 May 7;14:116. doi: 10.1186/1471-2180-14-116 (PMC4032360; doi:10.1186/1471-2180-14-116)

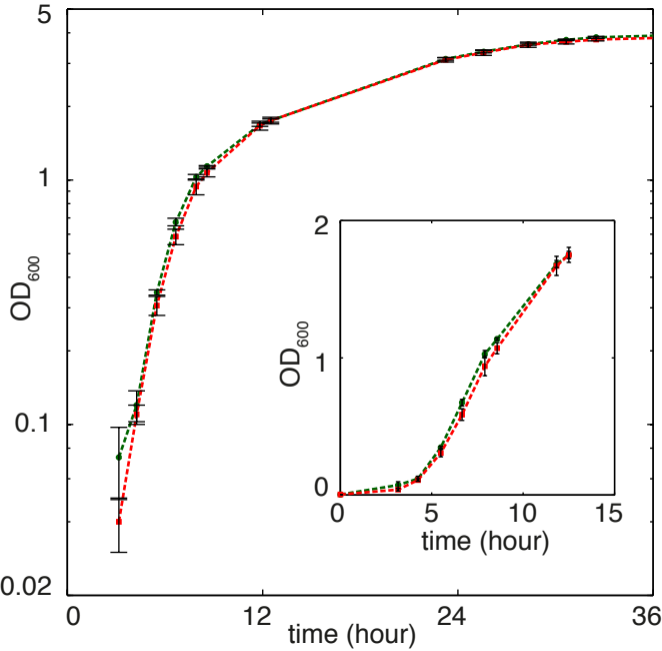

Supplement: Additional file 1 — Growth curves of strains JEK1036 and JEK1037 in bulk conditions. Growth curves are shown for strains JEK1036 (in green) and JEK1037 (in red), for each strain 3 independent cultures were grown in 200 ml LB in 500 ml flasks at 30°C. For each sample the OD600 was measured in triplicate and their average value was used. Error bars indicate sem. The inset shows the growth curve using linear y-scale for the first 15 hours. [file 1471-2180-14-116-S1.pdf]

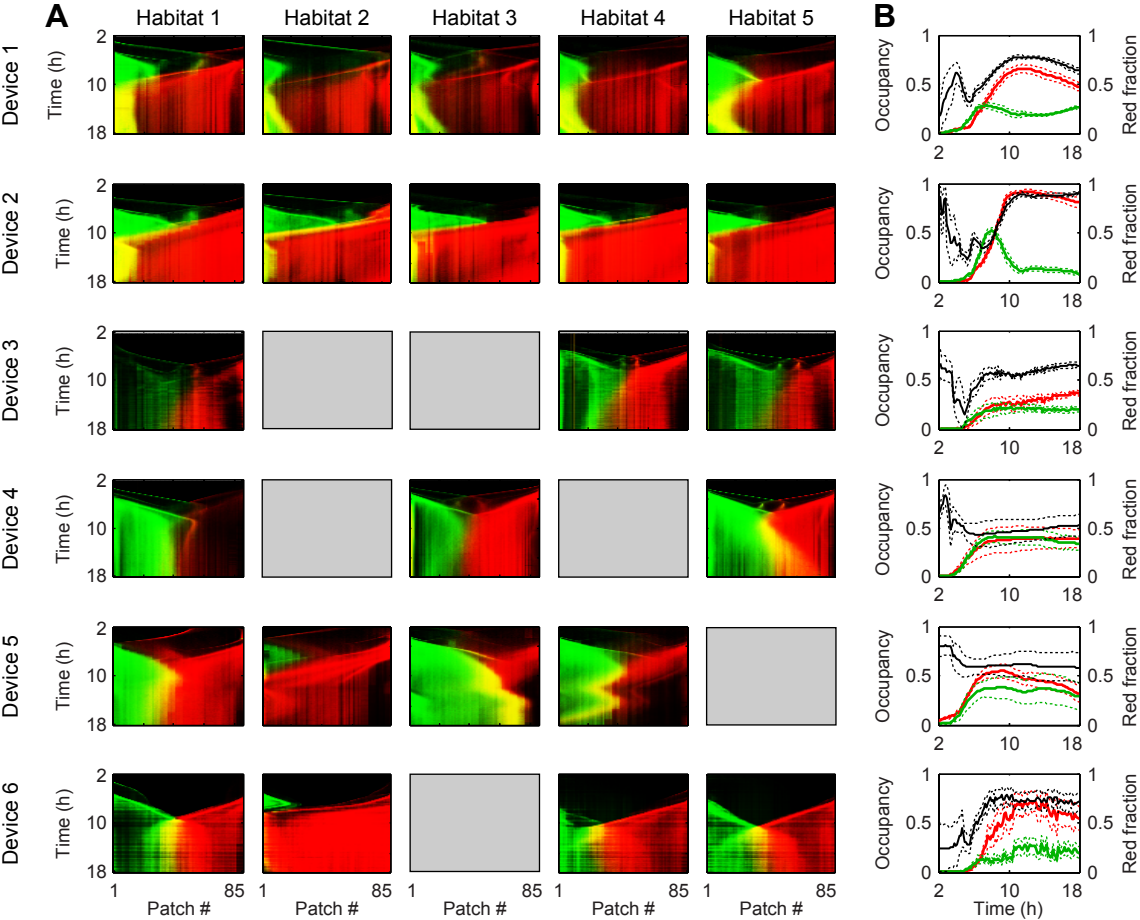

Supplement: Additional file 2 — Overview of all devices with separate inlets (type 1). (A) Each kymograph shows the average occupancy per patch in a single habitat. Kymographs for the five parallel habitats in a single device are shown next to each other. Note that all habitats on the same device are inoculated from the same culture set. (B) The device-wide averages of the occupancies of strains JEK1037 (R red) and JEK1036 (G green) and the red fraction (f r black) are shown as function of time. Dashed lines indicate mean ± sem. The red fraction (f r ) is calculated for each habitat as f r = r/(r + g), where r and g are the habitat-wide average occupancies of strains JEK1037 (red) and JEK1036 (green) respectively. Habitats where one (or both) of the strains failed to enter (e.g. when there is a constriction in one of the inlet channels) were excluded from the analysis and are shown as grey panels in this figure. [file 1471-2180-14-116-S2.pdf]

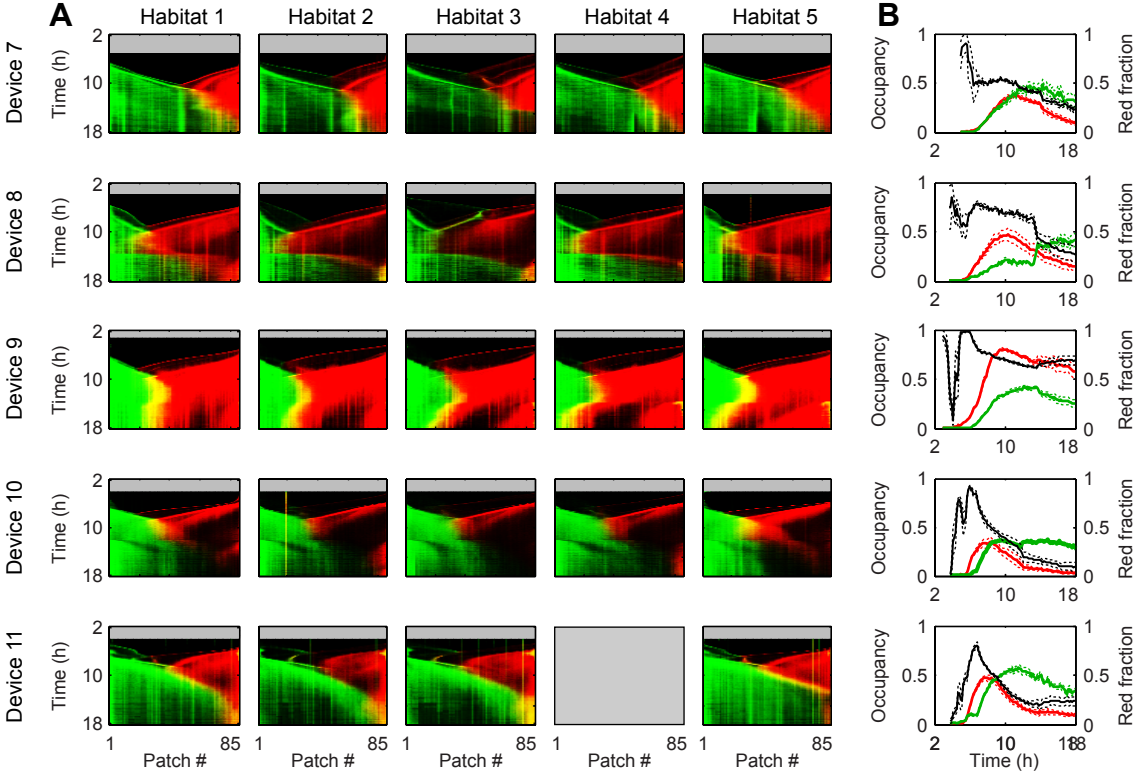

Supplement: Additional file 3 — Overview of all devices with a single inlet (type 2). (A) Each kymograph shows the average occupancy per patch in a single habitat. Kymographs for the five parallel habitats in a single device are shown next to each other. Note that all habitats on the same device are inoculated from the same culture set. (B) The device-wide averages of the occupancies of strains JEK1037 (R, red) and JEK1036 (G, green) and the red fraction (f r black) are shown as function of time. Dashed lines indicate mean ± sem. The red fraction (f r ) is calculated for each habitat as f r = r/(r + g), where r and g are the habitat-wide average occupancies of strains JEK1037 (red) and JEK1036 (green) respectively. Habitats where one (or both) of the strains failed to enter (e.g. when there is a constriction in one of the inlet channels) were excluded from the analysis and are shown as grey panels in this figure. Note that devices 10 and 11 were inoculated from the same initial cultures. [file 1471-2180-14-116-S3.pdf]

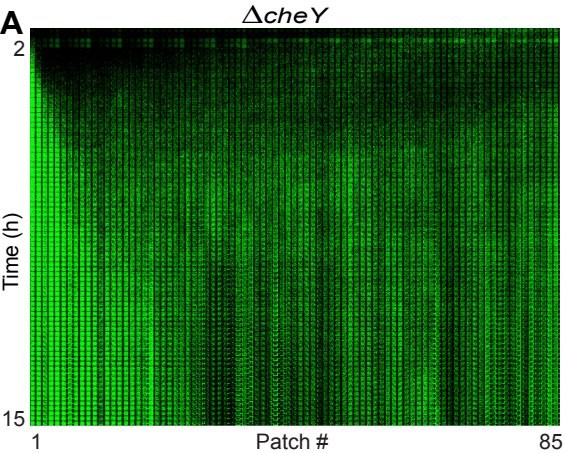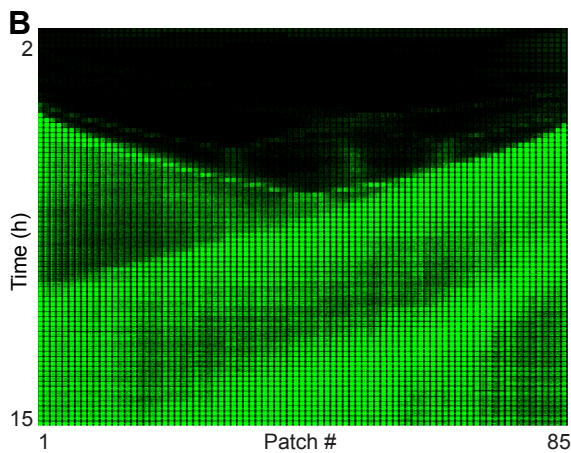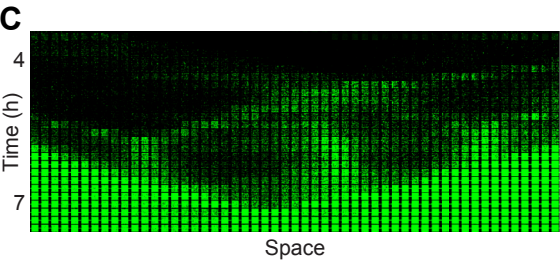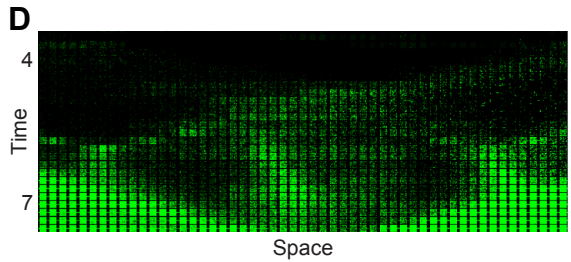

Supplement: Additional file 4 — Interactions between populations originating from the same initial culture. (A) Kymograph of fluorescence intensity for a type-1 device inoculated at both sides with the non-chemotactic, smooth-swimming, strain JEK1038 (ΔcheY). (B) Kymograph of fluorescence intensity for one habitat in a type-1 device that was inoculated at both sides with cells coming from the same initial culture of strain JEK1036. (C) Enlarged part of panel B. (D) Enlarged part of a different habitat in the same device as shown in panels B and C. [file 1471-2180-14-116-S4.pdf]

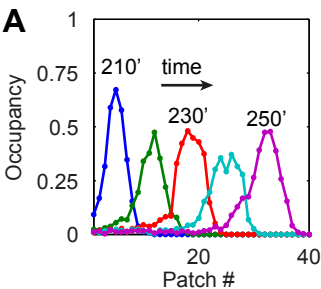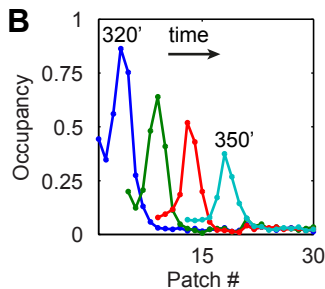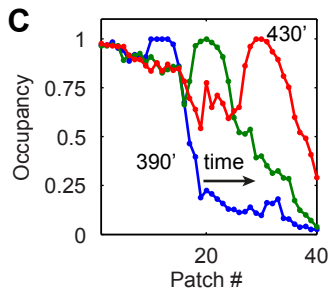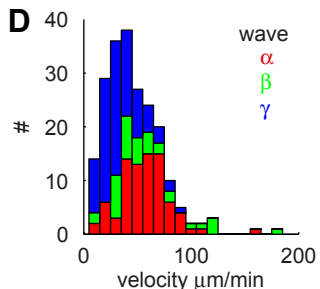

Supplement: Additional file 5 — Bacterial colonization waves in patchy habitats. (A) Wave profile of the α wave shown in Figure 1D, shown here as the area fraction occupied per patch (occupancy) as function of space, different lines show the profile for t = 210 min to t = 250 min in steps of 10 minutes. (B) Wave profile for the β wave shown in Figure 1D, different lines show the profile for t = 320 min to t = 350 min in steps of 10 minutes. (C) Wave profile for the γ wave and expansion front (F) shown in Figure 1D, different lines show the profile for t = 390 min to t = 430 min in steps of 20 minutes. (D) Distribution of wave velocities (of strains JEK1036 and JEK1037 combined) for α (red), β (green) and γ (blue) waves. [file 1471-2180-14-116-S5.pdf]

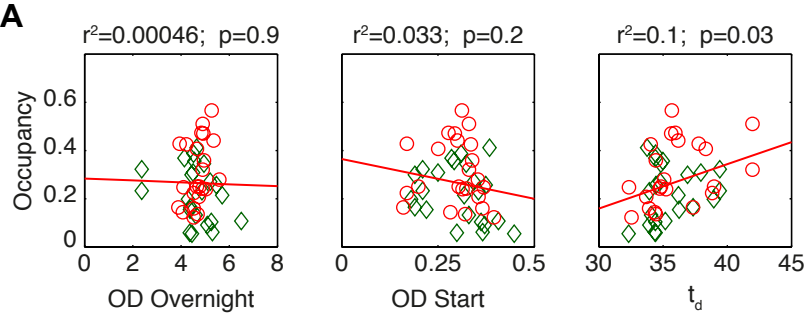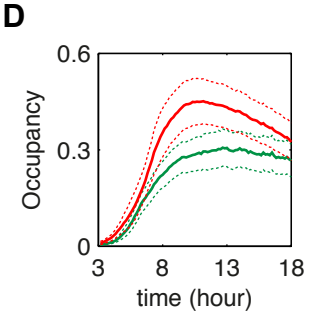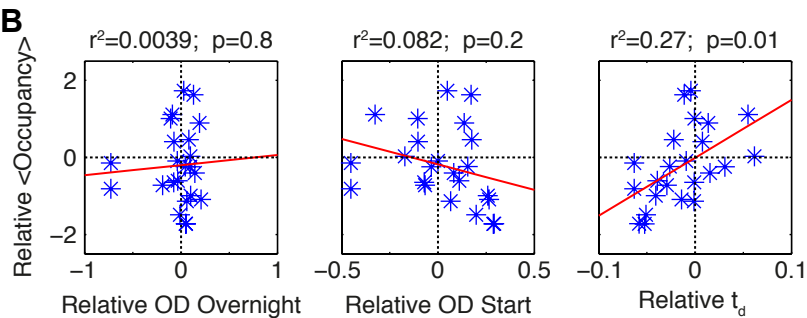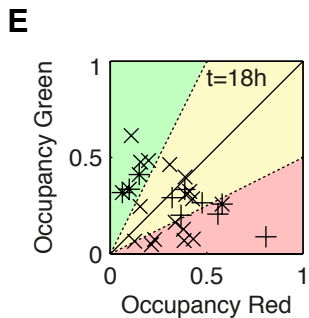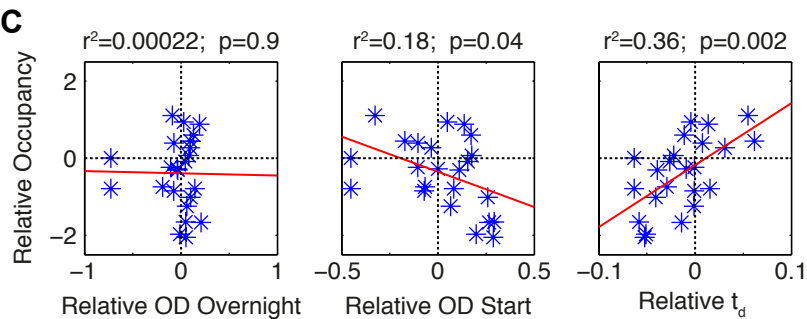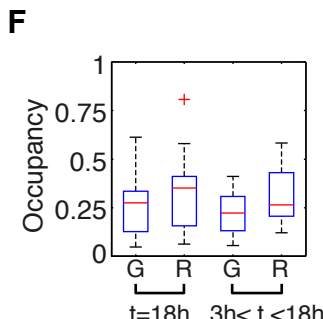

Supplement: Additional file 6 — Effects of the strain and the bulk growth parameters on the occupancy obtained in the habitats. (A-C) Relation between the occupancy obtained in the habitat and three bulk growth parameters: (i) OD overnight: the OD600 of the overnight culture; (ii) OD start: OD600 of the initial culture (iii): t d : the average doubling time of the initial culture during growth after back-dilution. Relative values are calculated for each culture-set by dividing the measurement for strain JEK1036 (green) by the corresponding measurement for strain JEK1037 (red) and taking the log of this ratio, i.e. as log[X(green)/X(red)], where X represents the measure of interest (A) Relation between bulk growth parameters and the occupancy at t = 18 h, for strain JEK1036 (green diamonds) and strain JEK1037 (red circles). (B) Relation between the relative occupancy averaged over the entire colonization process (i.e. 3 < t < 18 h) and the relative bulk growth parameters. (C) Relation between the relative occupancy at t = 18 h and the relative bulk growth parameters. Linear regression lines are shown in red, r2 values (of Pearson correlation) and the corresponding p-values are shown above each panel. (D-F) Comparison of the occupancy obtained in the habitat by strain JEK1036 (green) and JEK1037 (red). The data shown is based on all habitats of devices of types-1, 2 and 5. Measurements of habitats inoculated from the same culture set were averaged before combining them with data from other experiments. (D) Average occupancies of strains JEK1036 (green solid line) and strain JEK1037 (red solid line) as function of time, dashed lines indicate 95% confidence intervals. (E) Occupancy of strain JEK1036 plotted as function of the occupancy of strain JEK1037 at t = 18 h. Each point corresponds to the average occupancy obtained in the habitats inoculated from the same culture set. Symbols indicate the device type: plus-signs (+): type-1, stars (*): type-2, crosses (x): type-5. (F) Distribution of occupancie [file 1471-2180-14-116-S6.pdf]

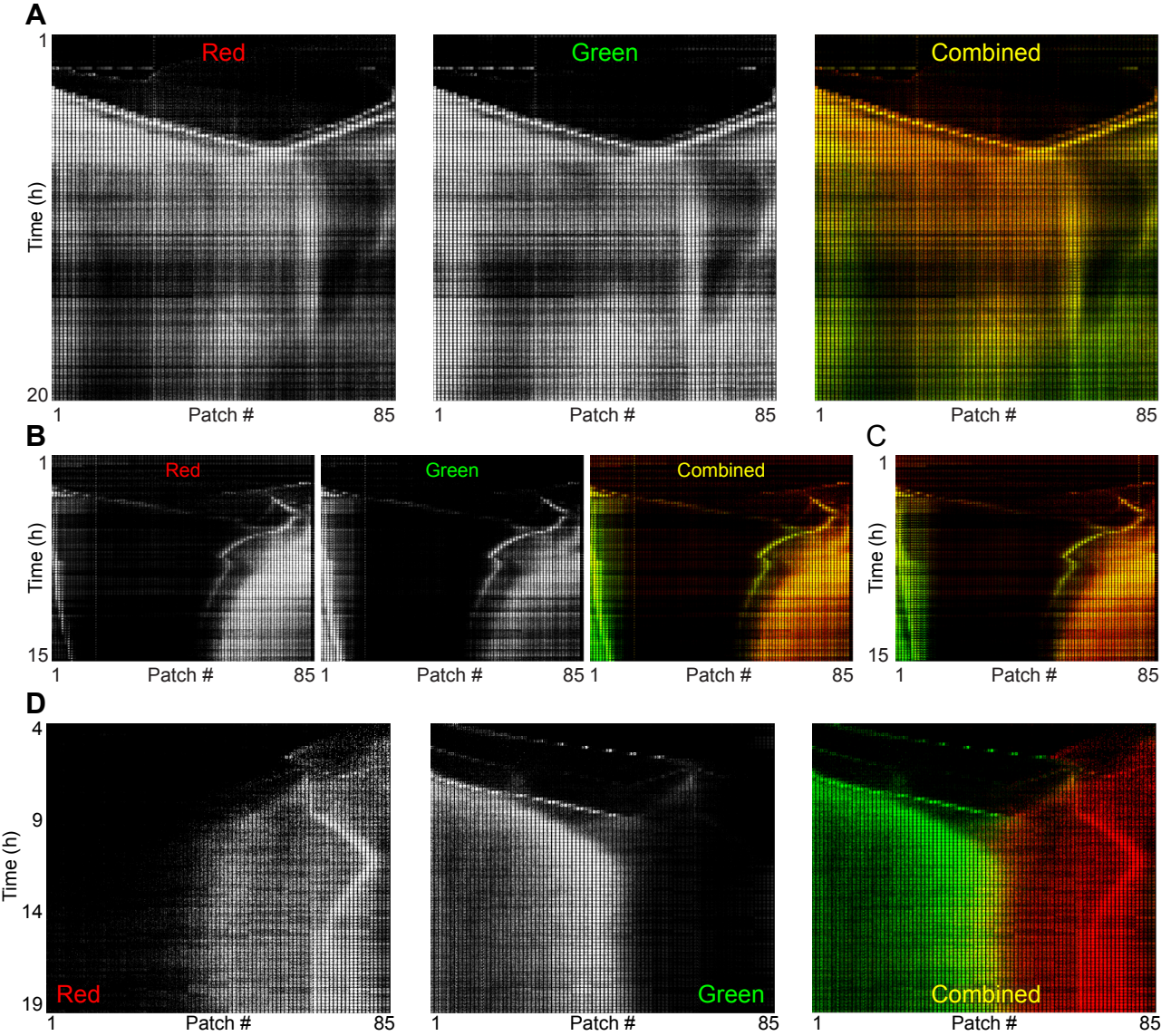

Supplement: Additional file 7 — Devices inoculated at both ends with a mixed culture of strains JEK1036 and JEK1037. (A) Kymographs of fluorescence intensity for a device with separate inlets (type 1; Figure 1A) inoculated at both ends with a single mixed culture of strains JEK1036 and JEK1037, with the kymograph of RFP (JEK1037) on the left, of GFP (JEK1036) in the middle and of the combined colors on the right. Note how the two strains remain mixed throughout the experiments, in contrast, the strains remain spatially segregated when inoculated from opposite sides of the habitat, as shown in panel D. (B) Kymographs of fluorescence intensity for a device with a single inlet (type 2; Figure 1B) inoculated at both ends with a single mixed culture of strains JEK1036 and JEK1037, with the kymograph of RFP (JEK1037) on the left, of GFP (JEK1036) in the middle and of the combined colors on the right. (C) Kymographs of fluorescence intensity for a different habitat in the same device as shown in panel B, inoculated at both ends with a single mixed culture of strains JEK1036 and JEK1037, note the similarity between the patterns in panels B and C. (D) As reference the kymographs are shown for the habitat shown in Figure 4A, with the kymograph of RFP (JEK1037) on the left, of GFP (JEK1036) in the middle and of the combined colors on the right. [file 1471-2180-14-116-S7.pdf]

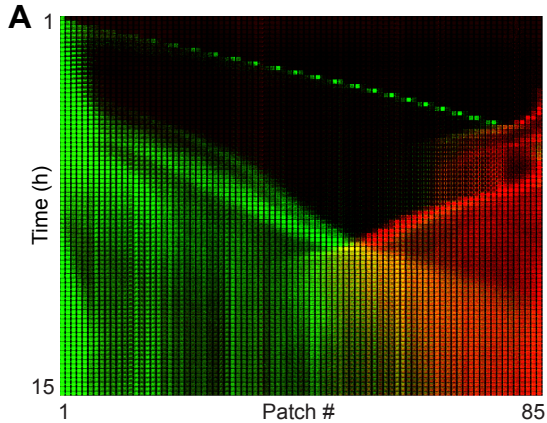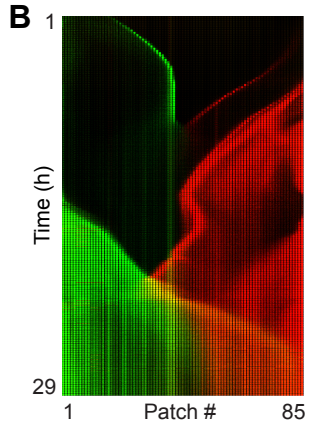

Supplement: Additional file 8 — Interactions between chemically coupled, but physically separated population. Kymographs are shown for two type-3 devices. The fluorescence intensities of the top and bottom habitat are superimposed: cells in the top habitat are shown in red and cells in the bottom habitat in green. Note that both habitats are inoculated from the same (JEK1036, green) culture, and that the bacteria in the upper and lower habitats are spatially confined to their own habitat. [file 1471-2180-14-116-S8.pdf]

**A**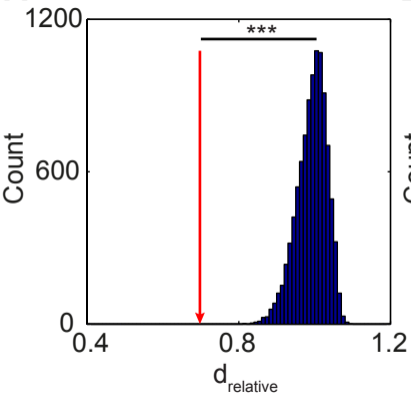**B**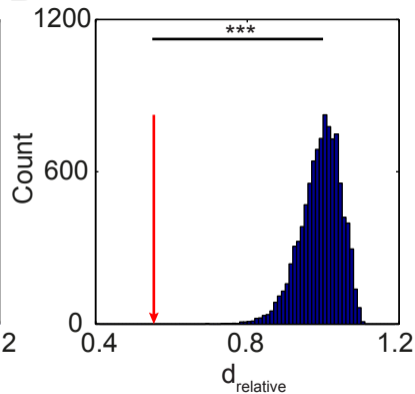**C**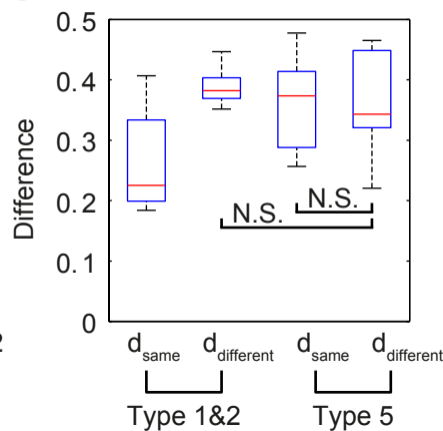

Supplement: Additional file 9 — Similarity between spatiotemporal patterns. Panels (A and B) show the results of a randomization test performed to assess the effect of the initial cultures on the degree of similarity between population distributions. For each habitat we calculated the ratio of the average difference in population distributions of habitats inoculated from the same cultures () relative to the average difference to all habitats inoculated from different cultures (): d relative =/. The red arrows indicate , obtained by averaging log[d relative ] over all habitats of a given device type. The blue distribution shows the values of obtained using 10.000 randomizations, where each population distribution was assigned to a randomly chosen habitat. Note that values of d relative were log transformed before averaging, the figure shows the back-transformed values. (A) Devices of type-1. (B) Devices of type 2. Note how in all cases the for the real dataset (in red) is much lower than the obtained from the randomized dataset (in blue). *** indicates p < 0.001. (C) Comparison of the degree of similarity observed in type-1 and 2 devices combined to that observed in devices of type-5. For both groups the differences between population distributions in habitats inoculated from the same culture set (d same ) and the difference between population distributions in habitats inoculated from different culture sets (d different ) is shown. Values of d same and d different obtained for habitats inoculated from the same culture sets were averaged together. N.S. indicates p > 0.05 in a Wilcoxon rank sum test (comparison of d different between type 1 and 5 devices) or Wilcoxon signed rank test (comparison between d same and d different for type 5 devices). [file 1471-2180-14-116-S9.pdf]

**A**  
1

Habitat 1

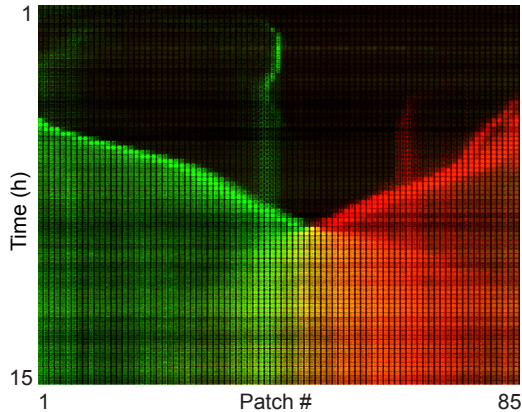

Habitat 2

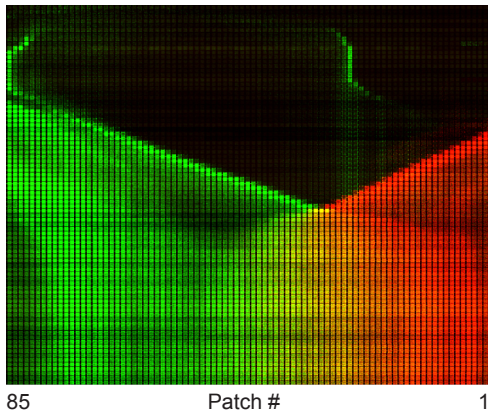**B**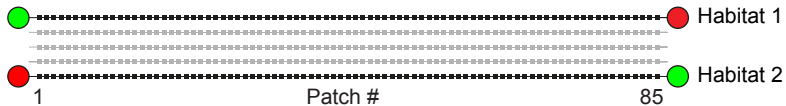

Supplement: Additional file 10 — Device type-4 where the two habitats where inoculated in reverse orientation. (A) Kymograph of fluorescence intensity for a device of type-4, where only the two outer most habitats are used. The orientation of inoculation was reversed for the two habitats, i.e. the red strain was inoculated from the right into habitat 1 and from left into habitat 2, see panel B. Note that the kymograph of habitat 2 is horizontally mirrored to reveal the similarity with habitat 1. (B) Schematic of the inoculation locations. [file 1471-2180-14-116-S10.pdf]

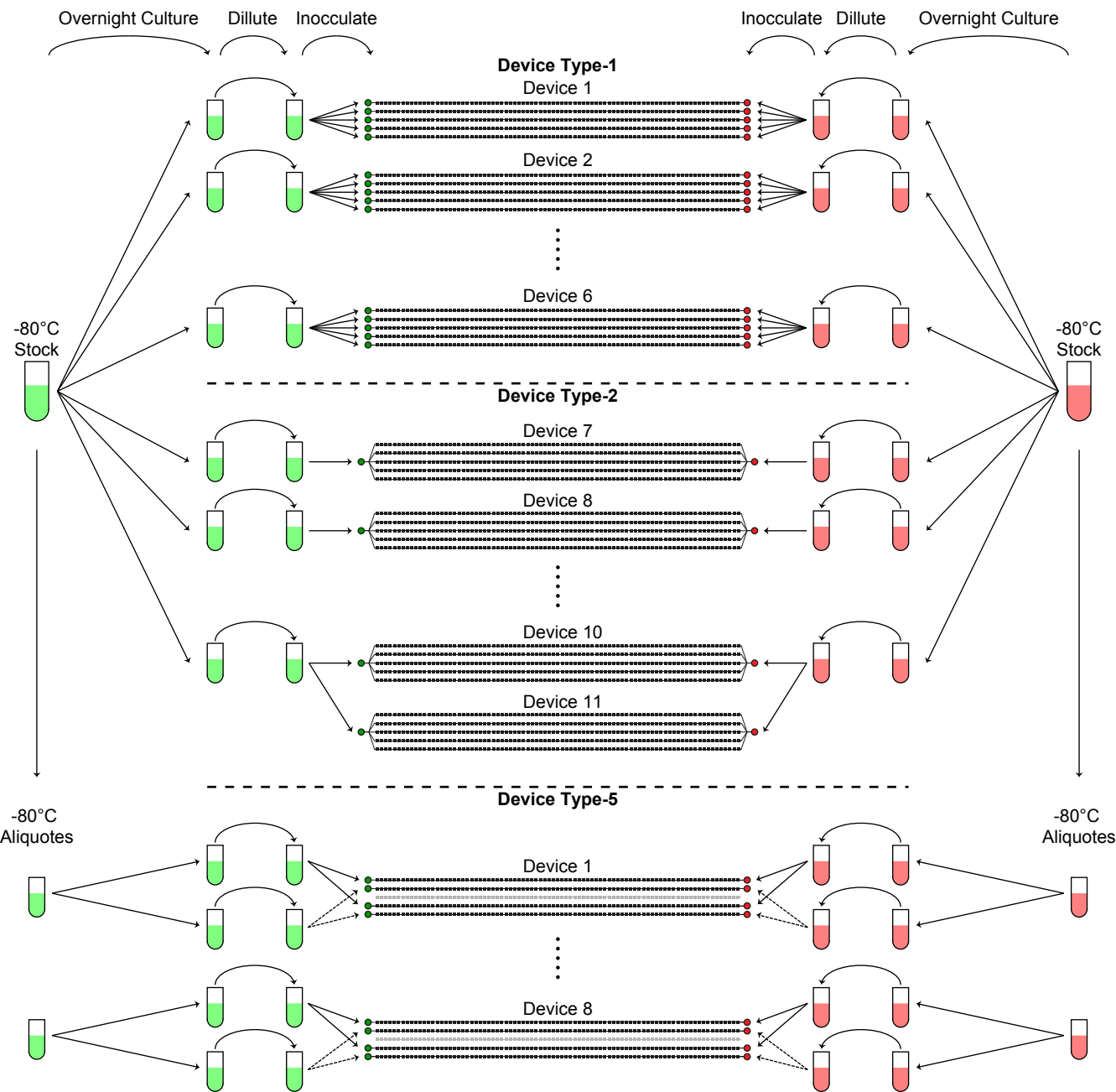

Supplement: Additional file 11 — Experimental Protocol. Protocol for the experiments using type-1 (top part), type-2 (middle part) and type-5 (lower apart) devices. Devices 10 and 11 (type-2) were imaged in parallel on the same microscope setup, after being inoculate from the same set of initial cultures. For devices of types 1 and 2 overnight cultures were started by taking a sample (of undefined volume) from a single −80°C stock for each strain, for devices of type-5 these same −80°C stocks (one for each strain) were split into aliquots and each overnight culture was started using a defined volume of a thawed aliquot. The following morning cultures were back-diluted 1:1000 to result in the initial culture with which the devices were inoculated. [file 1471-2180-14-116-S11.pdf]

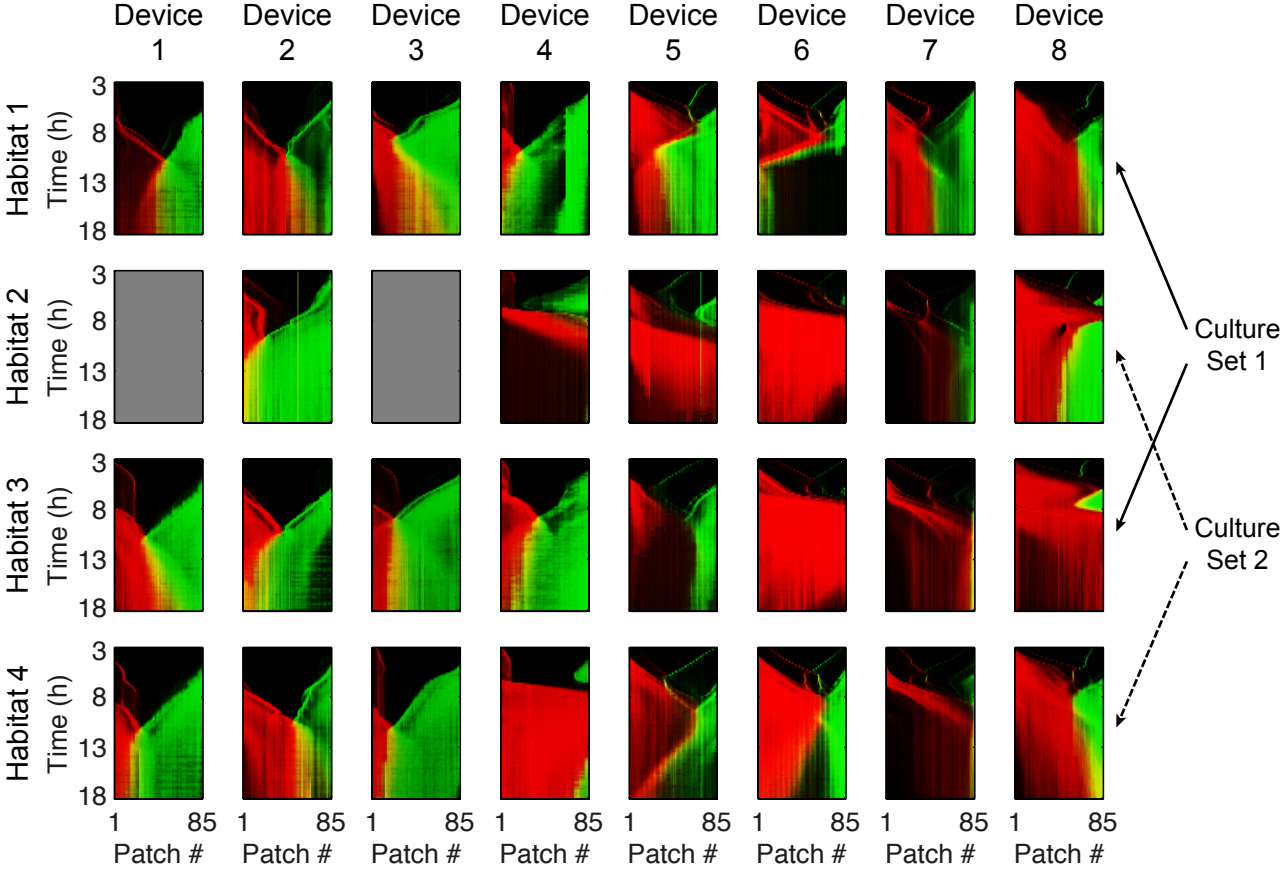

Supplement: Additional file 12 — Overview of all devices of type-5. Each kymograph shows the average occupancy per patch in a single habitat. Kymographs for the four parallel habitats in a single device are shown below each other. Note that devices were inoculated from two different sets of initial cultures: habitats 1 and 3 from culture set 1 and habitats 2 and 4 from culture set 2. Habitats where one (or both) of the strains failed to enter (e.g. when there is a constriction in one of the inlet channels) were excluded from the analysis and are shown as grey panels in this figure. [file 1471-2180-14-116-S12.pdf]
